# Bmi1 deficiency exacerbates hyperoxia-induced acute lung injury in mice

**DOI:** 10.3389/fphys.2025.1695456

**Published:** 2025-11-13

**Authors:** Helena Hernández-Cuervo, Ramani Soundararajan, Sahebgowda Sidramagowda Patil, Mason T. Breitzig, Matthew Alleyn, Venkata Ramireddy Narala, Richard Lockey, Lakshmi Galam, Narasaiah Kolliputi

**Affiliations:** 1 Department of Dermatology, Miller School of Medicine, University of Miami, Miami, FL, United States; 2 Department of Surgery, Morsani College of Medicine, University of South Florida, Tampa, FL, United States; 3 Anatomy and Cell Biology Department, University of Iowa, Iowa City, IA, United States; 4 Division of Epidemiology, Department of Public Health Sciences, Penn State College of Medicine, Pennsylvania State University, Hershey, PA, United States; 5 Division of Allergy and Immunology, Department of Internal Medicine, Morsani College of Medicine, University of South Florida, Tampa, FL, United States; 6 Department of Zoology, Yogi Vemana University, Kadapa, India; 7 Department of Molecular Medicine, Morsani College of Medicine, University of South Florida, Tampa, FL, United States

**Keywords:** BMI1, hali, hyperoxia-induced acute lung injury, mitochondria, ARDS (acute respiratory disease syndrome)

## Abstract

**Introduction:**

Administering high levels of oxygen is a life-sustaining measure in critically ill lung subjects. However, prolonged hyperoxia exposure increases reactive oxygen species (ROS) that exacerbate oxidative stress, mitochondrial dysfunction, respiratory failure, and cell death. Mitochondria play a critical role in hyperoxia-induced acute lung injury (HALI). The specific role of the Polycomb Repressive Complex 1 protein BMI1 (B cell-specific Moloney murine leukemia virus integration site 1) in mitochondrial damage remains unknown.

**Methods:**

*Bmi1* knockout mice (*Bmi1* ^−/−^) and their wild-type (*WT*) littermates were exposed to normobaric hyperoxia using oxygen concentrations of 95-100% for 48 h to assess BMI1 function in HALI. This research included the estimation of protein and gene expression, live mitochondria isolation, Oxygen consumption rate measurement, histomorphology analysis, capillary assessment, and dynamic lung function evaluation.

**Results:**

Mice lacking *Bmi1* versus *WT* exposed to hyperoxia exhibited hallmarks of human acute lung injury (ALI) such as increased lung permeability, alveolar edema, hemorrhage, interstitial thickening, and infiltration of immune cells; and alterations in lung mechanics, including increased elastance and decreased lung compliance.

**Discussion:**

*Bmi1* ^−/−^ mice exhibit increased mitochondrial damage, increased oxidative stress, and significant changes in protein markers related to mitophagy compared to *WT* mice. Our results indicate that *Bmi1* ^−/−^ mice are susceptible to HALI, and the damage increases in those mice compared with their *WT* littermates.

## 1 Introduction

B cell-specific Moloney murine leukemia virus integration site 1 (BMI1) is a proto-oncogene and member of the Polycomb Repressive Complex 1 (PCR1). This ubiquitous multi-functional protein regulates the cell cycle regulation and homeostasis, stem cell renewal, chromatin acetylation, development, and activation of the DNA damage response (DDR) pathway (Cho et al., 2013). The role of BMI1 is important in mitochondrial metabolism (Dias-Freitas et al., 2016; Bhattacharya et al., 2015). *Bmi1* null mice are small with low body weight and manifest neurologic abnormalities such as ataxia, tremors, and seizures (Van der Lugt et al., 1994). They have under-developed hematopoietic systems, reduced thymus, spleen, and liver size, and a failure of bone marrow cell production (Park et al., 2003). *Bmi1* null mice also have a reduced cellular and humoral immune response, decreased lymphopoiesis and myelopoiesis, high susceptibility to opportunistic infections, and alterations in bone morphology (van der Lugt et al., 1994).

BMI1 protein expression is necessary for cell renewal and the response to injury caused by oxidative stress in lung stem cells. BMI1 regulates different pathways. Upregulation of BMI1 is first associated with the overexpression of phosphatidylinositol 3 kinase (PI3K), Protein Kinase B (p-AKT), phosphorylated at Ser473, and mammalian target of rapamycin (mTOR); and second with the inhibition of phosphatase and tensin homolog (PTEN). The two activities regulate the PI3K/Akt-mTOR pathway in cardiac dysfunction and cardiac fibrosis in mice (Yang et al., 2019). BMI1 is a signaling protein related to mitochondrial function and autophagy-mediated necroptosis (a programmed cell death that triggers mitophagy) (Zhang et al., 2020; Shi et al., 2019; Dey et al., 2016; Park et al., 2003; Ryter and Choi, 2010; Aggarwal et al., 2016). *Bmi1* knockout mice manifest alterations in the electron transport chain (ETC) and increased production of mitochondrial reactive oxygen species (ROS). In mice, deficiency of BMI1 protein has been related with alterations in thymocyte maturation, the self-renewal capacity in stem cells and the reduction on lifespan (Bhattacharya et al., 2015; Liu et al., 2009; van der Lugt et al., 1994; Baughman and Mootha, 2006). Liu and collaborators (2009) shown how the deficiency of BMI1 expression cause mitochondrial disfunction in thymocytes and mice’s bone marrow cells and evidenced increased intracellular levels of reactive oxygen species. However, its role in acute lung injury (ALI) and acute respiratory distress syndrome (ARDS) is unknown.

ALI is characterized by an innate immune response, polymorphonuclear (PMN) cell migration, cytokine storm, pulmonary permeability, and death of lung epithelial cells and vascular endothelium. Injurious levels of oxygen give rise to hyperoxia-induced acute lung injury (HALI) which, after extended exposure, can lead to ARDS (Bhandari, 2008; Zaher et al., 2007). Exposure of lung cells to hyperoxia triggers a cascade of pathophysiological events, activating inflammation promoters, inducing mitochondrial damage, increasing ROS production and oxidative stress, triggering cell death and activating alveolar macrophages (generating more inflammatory cytokines), followed by PMN recruitment, loss of the alveolar-capillary barrier, increase in interstitium, and formation of a hyaline membrane (Dias-Freitas et al., 2016; Bhandari, 2008; Zaher et al., 2007).

Maintaining mitochondrial health and its bioenergetic metabolism is necessary for effective ALI treatment. Islam and colleagues, (2012) explored the relevance of mitochondria in ALI/ARDS. They demonstrated the process by which healthy mitochondria from cultured bone marrow-derived stromal cells (BMSCs) instilled into the lungs, migrate in vesicles to injured epithelial cells to repair the damage and protect against injury (Islam et al., 2012; Prockop, 2012). Another study demonstrated how transplanted mitochondria move from mesenchymal stromal cells through tunneling nanotube (TNT)-like structures, enhancing the phagocytic activity of macrophages in response to cell damage in ARDS (Jackson et al., 2016). A balance between fusion and fission proteins is required for mitochondrial integrity, avoiding mitophagy, and maintaining cell survival and stability (Tilokani et al., 2018; Yoo and Jung, 2018) playing a crucial role in ALI (Zhang et al., 2020).

The current investigation showed that Bmi1 expression decreased under hyperoxic conditions in wild-type (*WT*) mice and contributed to HALI-associated organ damage (Valiente-Alandi et al., 2016; Dong et al., 2014; Herrero et al., 2018a; Herrero et al., 2018b; Lee et al., 2016). In *Bmi1* ^−/−^ knockout mice was demonstrated that *Bmi1* genetic deletion is related to the worsening and severity of HALI.

## 2 Materials and methods

### 2.1 Experimental animals

Heterozygous *Bmi1* ^+/−^ mice of friends of leukemia virus B (FVB) background were obtained from The Jackson Laboratory (stock No: 024584, Allele Symbol: *Bmi1* ^*tm1Brn*^) and bred to obtain all the genotypes required for future experiments and colony maintenance. The University of South Florida Institutional Animal Care and Use Committee (IACUC) approved all animal experiments. The mice were housed in the USF Comparative Medicine facility in isolated cages, with a 12 h light-dark cycle at 22 °C ± 1 °C and fed a standard diet *ad libitum*. Knockout (*KO*) mice received hydrogel, and fat and protein-rich food *ad libitum* in addition to the standard diet. The mice colony was genotyped by TransnetYX® Inc., and experimental animals were used at 9 weeks for all the experiments. *Bmi1* gene transcript levels were confirmed at the beginning of the study using TaqMan RT-qPCR probes Bmi1 (Mm00776122_gH) and β-actin (Mm02619580_g1) to verify the absence of the transcript in *Bmi1* ^−/−^ mice (Supplementary Figure S1C). Both male and female mice (8–10 weeks old, n = 6–8 per group) were used for all experiments. A total of 83 *Bmi1* knockout mice (*Bmi1* ^−/−^) and 83 of their *WT* littermates were used in this study, being 58% females and 42% males.

### 2.2 *In vivo* hyperoxia model

*Bmi1* knockout mice (*Bmi1* ^−/−^) and their *WT* littermates were used for experimentation at 9 weeks old. Mice were exposed to normoxic: NO (21% O_2_) or hyperoxic: HO (O_2_ >95%) conditions in an airtight sealed Plexiglass chamber (70 × 50 × 50 cm) from Coy Laboratory Products, for 48 h to induce HALI with continuous O_2_ monitoring and CO_2_ maintained <0.5%. Oxygen levels were monitored using a ProOx 110 controller (BioSpherix, NY). After treatment, the mice were anesthetized by intraperitoneal injection with a dose of 0.01 mg/kg of Ketamine and 0.001 mg/kg of Xylazine (stock solution was made by mixing 10 mg/mL of Ketamine and 1 mg/mL of Xylazine in saline solution at 0.09%). Following anesthesia, the mice were cervically dislocated before proceeding with the lung perfusion and collection. The lungs were perfused with 5 mL of sterile phosphate-buffered saline (PBS) and either snap-frozen in liquid nitrogen, fixed in 4% paraformaldehyde for histology, or immediately processed for live mitochondria isolation.

### 2.3 Protein isolation

Lung tissue was stored in liquid nitrogen. Lung protein was obtained by tissue pulverization using a liquid nitrogen-cooled steel pulverizer, suspended in protein isolation buffer (150 mM NaCl, 50 mM Tris, and 0.5% NP40, pH 7.4, supplemented with protease and phosphatase inhibitors 1:100), followed by grinding in disposable plastic homogenizers (BioMasher® II Micro Tissue Homogenizers, 1.5 mL tube with a pestle, DWK Life Sciences (Kimble) Millville, NJ) and thermal shock induced by freezing and thawing three times. The lung lysates were sonicated for 5 min (15 s pulses with 10 s intervals) at 50% amplitude in a Qsonica Q700 sonicator (Newton, CT). The lysates were then centrifuged at 21,000×*g* for 15 min at 4 °C, the supernatant collected and stored in low-binding protein tubes at −80 °C. Protein was quantified using a BCA protein assay kit following the manufacturer’s recommendations (Thermo Fisher Scientific, Inc. #23225, Pierce, Rockford, Waltham, MA).

### 2.4 Western blot analysis

Protein expression was analyzed by loading 10 μg of lung lysate from *WT* and *Bmi1* ^−/−^ mice exposed to NO or HO conditions on 10% or gradient SDS-PAGE (4%–20%). The proteins were transferred to the polyvinylidene difluoride (PVDF) membrane, blocked in 5% BSA in TBST for 1 h at room temperature, then incubated with primary antibody overnight at 4 °C. After washes, a secondary antibody (anti-rabbit HRP or anti-mouse HRP) was added and incubated for 1 h at room temperature, followed by serial washes. Proteins were visualized with Enhanced chemiluminescence (ECL) Kwik quant solution (Kindle Biosciences, Greenwich, CT), ECL, or Femto (Thermo Fisher Scientific, Inc. #32209 and #34095, Pierce, Rockford, Waltham, MA). Expression of mitochondrial fusion proteins: Mitofusin and mitochondrial dynamin-like GTPase (OPA1), fission protein (Drp1), and proteins involved in mitophagy and the PI3K pathway (Pink1, Parkin, Akt, Pten) was determined to establish the effect of *Bmi1* depletion.

Proteins were quantified using ImageJ (NIH, Bethesda, MD) and normalized to β-actin (antibodies and Western blot conditions are shown in Supplementary Table S1).

### 2.5 Live mitochondria isolation and mitochondrial proteins

Live mitochondria were isolated from the lungs by modifying the protocol published by Hartwig et al., 2015; Hartwig et al., 2015). Mice were euthanized, and whole-body perfusion was completed with 5 mL of sterile PBS. The lungs were kept on ice in homogenization buffer (HB) containing sucrose, EGTA, mannitol, DTT, NaCl, and KCl. Next, lung tissue was cut into small pieces and ground 10 times in a plastic disposable homogenizer containing 500 μL of HB (BioMasher® II Micro Tissue Homogenizers, 1.5 mL tube w/pestle, DWK Life Sciences, Kimble).

The homogenate was passed through a syringe with a 27-gauge needle 10 times and centrifuged at 666 *g* for 15 min at 4 °C. The supernatant was transferred to a 2 mL tube, and the volume brought up to 1.8 mL with HB and centrifuged at 11,000×*g* 4 °C for 15 min. The decanted supernatant containing cytosolic proteins was stored at −80 °C; the pellet was washed with HB by pipetting carefully, then it was resuspended in 1 mL of HB and centrifuged at 11,000×*g* for 15 min at 4 °C. The supernatant wash was discarded, and the pellet resuspended in 1 mL of HB. The suspension was overlaid on a 2, 1.5, and 1 M sucrose gradient prepared in HB (in 13.2 mL open-top thin-wall ultra-clear tubes, Beckman Coulter. Inc. Brea, CA) and centrifuged at 85000 *g,* 4 °C for 1 h without brake (Optima XPN-90 Ultracentrifuge with SW-41 Ti Swinging-Bucket Rotor, Beckman Coulter, Indianapolis, IN). The mitochondrial fraction was obtained from the middle layer and resuspended in cell growth medium for the oxygen consumption rate (OCR) measurement.

### 2.6 Measurement of OCR

Oxygen consumption rates of live mitochondria were determined using a Seahorse XFe96 Analyzer (Seahorse Biosciences, North Billerica, MA). Mitochondria were collected and stained with MitoTracker™ Green (Thermo Fisher Scientific, #M7514 Pierce, Rockford, Waltham, MA) at a final concentration of 5 μM for 5 min. The number of particles was quantified in a hemocytometer using a 40× objective and Olympus BX43 fluorescence microscope with an Olympus DP21 camera. One million particles were dissolved in 30 μL of Seahorse-Agilent pH 7.4 RPMI medium (supplemented with L-glutamine, glucose, and sodium pyruvate) and seeded in 96 well Seahorse-Agilent plates, centrifuged for 2 min at 200×*g* without brake, followed by a 1 h incubation in a CO_2_-free incubator. Then, 150 μL of Seahorse pH 7.4 RPMI medium was added to each well, and the OCR was measured by a Agilent Seahorse XF96 Analyzer. Data were analyzed using Seahorse Wave Desktop Software for XF Cell Mito stress test (Santa Clara, CA).

### 2.7 Histology

Left lungs were fixed in 4% PFA, processed, and sectioned by the Moffitt Cancer Center Tissue Core. Tissue sections stained with hematoxylin and eosin (H&E) were analyzed using 20 and 40 objectives on a Keyence BZ-X710 microscope to obtain an acute lung injury score (ALIS). Parameters for ALIS included the presence of peribronchiolar infiltrates, hemorrhage, cellular infiltration into alveoli (leucocyte or aggregation of neutrophils), increased interstitial thickening, and alveolar edema. Scores from zero to four were assigned as follows: (0) normal lung without damage, (1) mild damage <25% lung involvement; (2) moderate damage, 25%–50% lung involvement; (3) severe damage, 50%–75% lung involvement; and (4) very severe damage, >75% of the lung (Sue et al., 2004; Jain et al., 2007; Jain et al., 2008; Belperio et al., 2002; Christofidou-Solomidou et al., 2002). The ALIS was conducted blindly by three team members, and scores were individually collected, averaged, and analyzed.

### 2.8 Bronchoalveolar lavage fluid (BALF) analysis and cell counting

BALF was collected by instillation of 3 mL of DPBS (1 mL three times) into the lung via tracheal intubation using a peripheral 20G IV catheter for *WT* or 22G catheter for *Bmi1* ^*−/−*^ mice. BALF was centrifuged at 400 *g* at 4 °C for 10 min. The supernatant was transferred to a clean tube, stored at −80 °C. The pellets containing the cells were resuspended in 1 mL of DPBS. Total protein concentration in BALF was measured with a BCA protein assay following the manufacturer’s recommendations (Thermo Fisher Scientific, Inc., catalog 23225, Pierce, Rockford, Waltham, MA). The total cell number was estimated by counting in a hemocytometer using a 40× objective on an Olympus BX43 microscope and Olympus DP21 camera. For differential cell counting, 200 µL of cellular suspension was layered onto glass slides using a cytospin cytocentrifuge at 800 rpm for 5 min. Slides were stained with Kwik-Diff™ (ThermoFisher Scientific, Inc., catalog 9990700, Waltham, MA, United States) by total immersion for 10 s in a fixative solution, 10 s in solution I, and 7 s in solution II, and air-dried at room temperature. The cells were counted using an Olympus BX43 microscope (Han and Ziegler, 2013; Shaik et al., 2015; Hernández-Cuervo H et al., 2022).

### 2.9 Capillary leak assessment

Evans blue dye (EBD; Sigma-Aldrich) used to determine the effect of *Bmi1* deletion on alveolar permeability and cell infiltration. The mice were weighed and injected into the tail vein with EBD (stock solution prepared at 0.5% (0.005 mg/μL) in PBS) at 50 mg/kg (10 μL of EBD 0.5% per gram of body weight). After 30 min, the animals were euthanized and BALF was collected, infiltrating cells counted and protein concentration was measured (Sue et al., 2004; Reddy et al., 2012; Wick et al., 2018; Narala et al., 2018). The lungs were excised, photographed, and frozen in liquid nitrogen. Lung capillary permeability was assessed in the BALF by measuring the EBD absorbance at 630 nm in a spectrophotometer.

### 2.10 Dynamic lung function measurements

Technical personnel from the Division of Comparative Medicine anesthetized and intubated the mice, and completed lung function measurements followed by euthanasia. *WT* and *KO* mice were exposed to NO or HO conditions (n = 6 mice per group). The animals received an intraperitoneal injection of dexmedetomidine (0.5 mg/kg) to induce sedation and reduce anxiety prior to intubation, followed by administration of the anesthetic (100 mg/kg ketamine+10 mg/kg xylazine). Each catheter was calibrated prior to use; 20G and 22G catheters were used for *WT* and *KO* mice, respectively. The mice were intubated after catheter calibration and maintained on isoflurane (3%–4% induction, 1%–2% maintenance/inhalation) delivered with oxygen using a calibrated vaporizer.

Lung function studies began with a “Deep Inflation” to standardize lung volume and verify the catheter’s placement. Baseline airway compliance and resistance were measured in triplicate (SnapShot-150, Quick Prime-3, and pressure-driven perturbation); the protocol was repeated twice (Verjans et al., 2018; Khadangi et al., 2021). All parameters, respiratory system elastance (Ers), tissue elastance (H), static compliance (Cst), tissue damping (G), Newtonian resistance (Rn), respiratory system resistance (Rrs), and inspiratory capacity (IC), were measured using a piston ventilator (FlexiVent, SCIREQ Inc., Montreal, Canada).The mice were placed on a circulating warm water pad to maintain body temperature during the procedure.

### 2.11 Statistical analysis

The normoxia/hyperoxia experiments were performed a minimum of two times. Data normality was assessed using the Shapiro-Wilk test prior to parametric analysis. The data were expressed as mean ± SE and evaluated using IBM SPSS Statistics (Version 26.0 Armonk, NY). Data were analyzed using ANOVA or two-way MANOVA followed by Tukey’s *post hoc* test for normally distributed data. A *p < 0.05* was considered statistically significant. Adobe Photoshop 2020 was used for assembling figures of high-resolution (600 dpi) original images.

## 3 Results

### 3.1 Phenotypic characterization of *Bmi1* ^−/−^ mice

*Bmi1* knockout mice weresmaller with lower body weight than WT littermates (Supplementary Figure S1A,B), and displayed ataxia, and impaired motor development.

### 3.2 Hyperoxia induces a decrease in Bmi1 protein levels in *WT* mice

**FIGURE 1:**
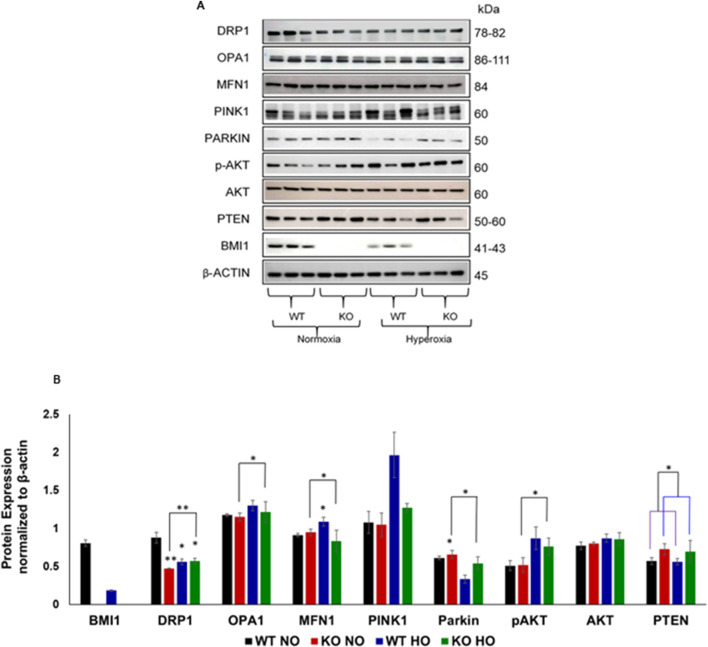
Mitochondrial protein expression in wild-type (*WT*) and Bmi1^−/−^ mice (*KO*). **(A)** Western blot analysis for mitochondrial dynamics and mitochondrial regulatory proteins in lung lysates from *WT* and *KO* mice after exposure to normoxia (NO) and hyperoxia (HO) conditions. **(B)** Densitometric analysis of Western blot normalized to β-actin. n = 6 mice per group, mean ± SEM; * p-value ≤0.05, ** p-value ≤0.01, ***p-value ≤0.001.

Bmi1 protein levels were determined by Western blotting to ascertain whether hyperoxia has any effect on Bmi1 expression. *WT* mice were initially subjected to normoxia or hyperoxia for 48 h Bmi1 protein expression was significantly decreased (p-value = 0.000282) after 48 h of HO exposure in *WT* mice. (Figure 1A). As a result of this finding, we sought to establish whether Bmi1 was required for HALI response.

### 3.3 Changes in protein expression in *Bmi1* ^−/−^ mice

The expression of several proteins was evaluated in the lung lysate isolated from *WT* and *Bmi1* ^−/−^ mice after NO and HO exposure (Figures 1A,B). Fission protein Drp1 (dynamin like-1 protein) expression in *Bmi1* ^−/−^ mice exposed to NO or HO and in *WT* mice exposed to HO was significantly decreased. Of the proteins analyzed by Western blot, only Drp1 showed a deleterious additive effect between the injury caused by HO and the deletion of *Bmi1* (Table 1). There were significant differences in Mitofusin1 and OPA1 between *WT* and *Bmi1* ^−/−^ mice fusion protein expression under HO but not in NO. Pink1, Parkin, and pAkt (Ser473) protein expression was affected by HO but not by the deletion of *Bmi1*.

**TABLE 1:** Summary of *p-values* for two-way MANOVA for protein expression levels in *WT* and *Bmi1* ^−/−^ mice (normalized data against β-actin).

Independent variable	Dependent variable							
	DRP1	OPA	MFN1	Pink1	Parkin	pAKT	AKT	PTEN
O_2_ Concentration	0.044	0.276	0.705	0.017	0.008	0.025	0.204	0.680
Genotype	0.002	0.518	0.203	0.086	0.056	0.661	0.927	0.011
O_2_* Genotype	0.002	0.726	0.105	0.110	0.182	0.604	0.745	0.836

The tumor suppressor protein Pten was inversely regulated by Bmi1 expression, showing increased expression in *Bmi1* knockout mice compared to *WT* mice in both NO and HO conditions. Comparison of protein expression between genotypes and oxygen exposure and multivariate analyses are shown in Figure 1B and Table 1, respectively.

### 3.4 *Bmi1* depletion interferes with mitochondrial respiration

**FIGURE 2:**
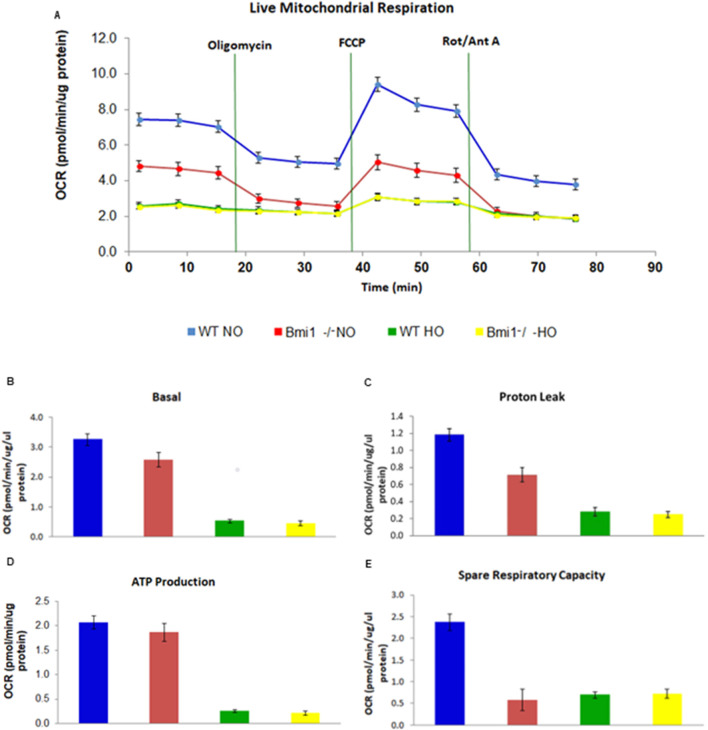
Oxygen Consumption Rate in live mitochondria. **(A)** Live mitochondrial respiration **(B)** Basal respiration **(C)** Proton leak **(D)** ATP production **(E)** Spare respiratory capacity. All parameters were expressed in pmol/min/μg of protein. Mean ± SEM. Two-way ANOVA *p-value ≤0.05, ** p-value ≤0.01, ***p-value≤0.001 all groups were compared vs. *WT* NO n = 6.

To establish if *Bmi1* depletion interfered with mitochondrial function, OCR experiments were performed on live mitochondria isolated from pulmonary tissue. Respiratory values were the highest in mitochondria derived from *WT* mice under NO (Figures 2A,B). Mice exposed to normoxic conditions, exhibited basal respiration of approximately 3.0 pmol/min/μg protein, while mice exposed to hyperoxic conditions was less than 1.0 pmol/min/μg. Similarly, ATP production decreased in hyperoxia conditions irrespective of genotype (Figure 2D). The proton leak and spare respiratory capacity were likewise affected by both the hyperoxia-induced injury and genotype (Figures 2C,E). The oxygen consumption rate was significantly higher in mitochondria isolated from the lungs of *WT* mice in NO versus HO conditions or *Bmi1* ^−/−^ mice under NO or HO, exhibiting a quiescent phenotype (Figure 2A).

### 3.5 *Bmi1* genetic deletion exacerbates hyperoxia-induced acute lung injury

Histological analysis of hematoxylin and eosin (H&E) stained lung sections from *WT* and *Bmi1* ^−/−^ mice exposed to NO and HO was performed to determine the relevance of *Bmi1* genetic deletion on lung structure. Blinded assessment of stained sections were examined by light microscopy to determine the ALIS. The lungs of *WT* mice exposed to 48 h of hyperoxia showed mild to moderate damage (ALIS = 1.92) including increased alveolar edema, alveolar wall thickening, and infiltration of immune cells (Table 2).

**TABLE 2:** Acute lung injury score (ALIS). Components of ALIS were evaluated for *WT* and *Bmi1* ^*−/−*^ mice exposed to normoxia and hyperoxia. Mean ± SEM.

Genotype	Components				
	Peribronchiolar infiltration	Hemorrhage	Immune cell infiltration	Interstitial thickening	Alveolar edema
WT NO	0.47 ± 0.13	0.07 ± 0.07	0.20 ± 0.12	0.67 ± 0.29	0.27 ± 0.18
*Bmi1* ^*−/−*^ Normoxia	1.80 ± 0.31	1.20 ± 0.31	2.20 ± 0.20	2.00 ± 0.12	1.73 ± 0.52
Wildtype Hyperoxia	1.47 ± 0.07	1.73 ± 0.24	2.60 ± 0.12	2.00 ± 0.12	1.80 ± 0.12
*Bmi1* ^*−/−*^ Hyperoxia	2.93 ± 0.41	2.60 ± 0.31	3.07 ± 0.18	2.93 ± 0.35	2.73 ± 0.18

**FIGURE 3:**
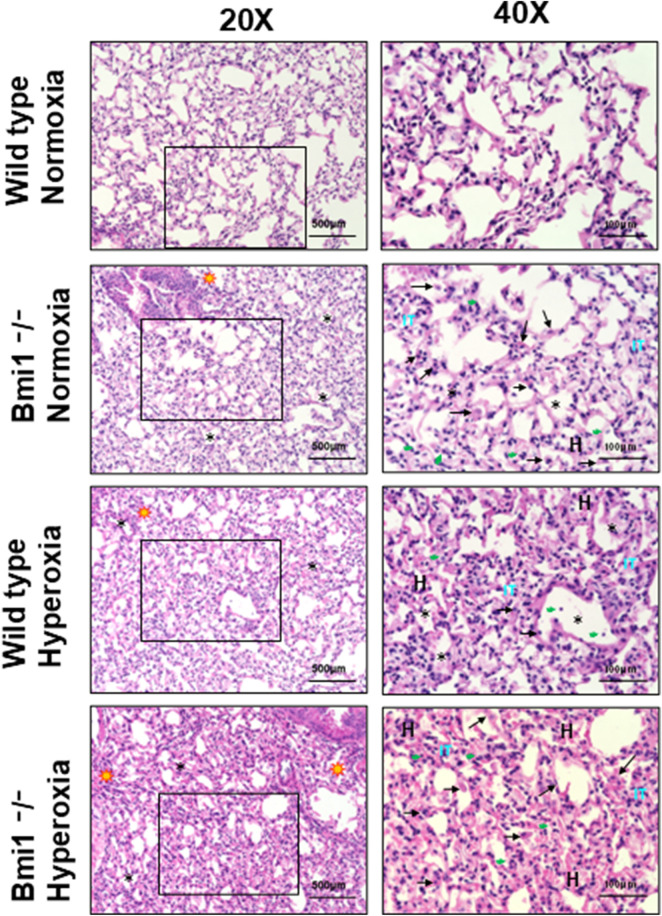
Acute lung injury score in wild type and Bmi1^−/−^ mice exposed to normoxia and hyperoxia. Acute lung injury parameters were evaluated in 5 randomized independent fields of 3 mice per each group, bars show mean ± SEM, * p-value ≤0.05, ** p-value ≤0.01, ***p-value ≤0.001 n = 6.

*Bmi1* ^*−/−*^ mice under NO conditions had moderate damage with an ALIS <2, while mice with the same genetic background exposed to 48 h of hyperoxia exhibited severe lung damage involving 50%–75% of the area analyzed (Table 2; Figure 3).

**FIGURE 4:**
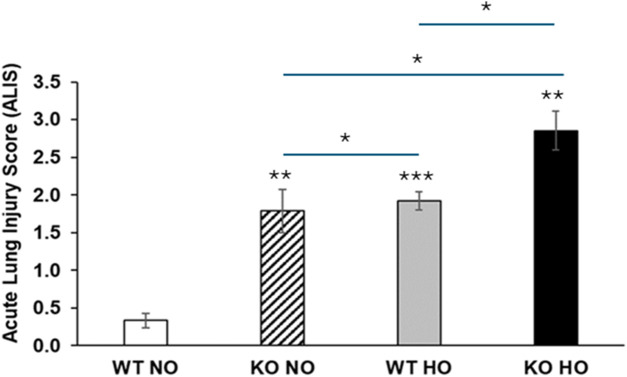
*Bmi1* genetic deletion exacerbates HALI. Representative photomicrographs of H&E-stained lung section from *WT* and *Bmi1* ^*−/−*^ mice exposed to normoxia and hyperoxia. Acute lung injury parameters were evaluated in 5 randomized independent fields of 3 mice per group. Black arrows show macrophages, green arrowheads indicate infiltrated immune cells, H in red represents hemorrhage areas, IT (clear blue) shows interstitial thickening, * designates alveolar edema and yellow stars are in areas with peribronchial infiltrates. Scale bar 100-500 μm, the inset (black box) is magnified.

Histological analysis revealed a deleterious effect caused by the genetic deletion of *Bmi1*. Although it is known that hyperoxia induces lung damage in *WT* mice, the damage was more pronounced in *Bmi1* ^*−/−*^ mice (Figure 4).

### 3.6 Lung injury determined by immune cell infiltration and alveolar permeability was increased in *Bmi1* ^*−/−*^ mice exposed to hyperoxia

Nine weeks old *Bmi1* ^*−/−*^ mice and their wild-type littermates were exposed to normoxia and hyperoxia for 48 h to evaluate if BMI1 plays a role in HALI, and the effect of *Bmi1* deletion on alveolar permeability and cell infiltration, BALF was collected, infiltrating cells counted and protein concentration was measured; the alveolar permeability to Evans blue dye (EBD) was determined.

**FIGURE 5:**
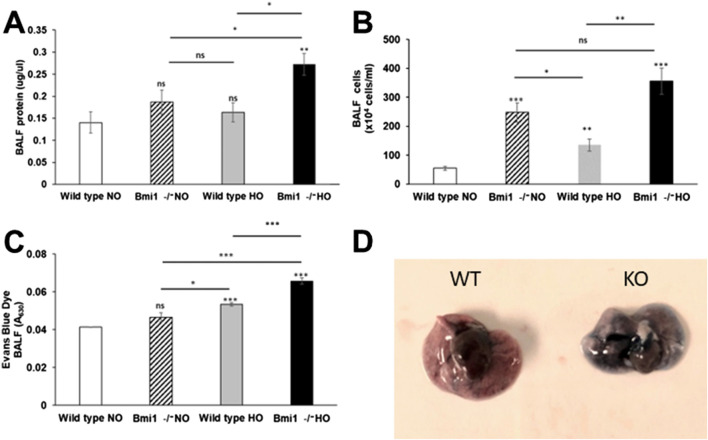
Analysis of lung permeability. *WT* and *Bmi1* ^*−/−*^ mice were exposed to normoxia and 48 h of hyperoxia and Bronchoalveolar lavage fluid (BALF) was collected. **(A)** Cells infiltration in BALF, **(B)** Protein quantification in BALF, **(C)** Alveolar permeability measured by EBD in BALF, **(D)** Comparison of lungs extracted from mice exposed to hyperoxia. Data represented as Mean ± SEM; for n = 6, * p-value ≤0.05, ** p-value ≤0.01, ***p-value ≤0.001.

*Bmi1* deletion promoted the infiltration of immune cells in BALF, and cell migration increased significantly after 48 h of hyperoxia (p < 0.001) (Figure 5A). Protein concentration in BALF was twice as high for *Bmi1* ^*−/−*^ versus *WT* mice exposed to hyperoxia (0.27 μg/μL) than in *WT* mice in normoxia (0.14 μg/μL) (Figure 5B). Extravasation of EBD into the alveolar space and collection in the BALF confirmed that the permeability of the alveolar was increased in *Bmi1* ^*−/−*^ mice. EBD permeability, measured by absorbance at 630 nm, was significantly higher in mice exposed to hyperoxia (*WT* and *KO*) with *Bmi1* ^*−/−*^ mice exhibiting a higher value (Figures 5C,D).

**FIGURE 6:**
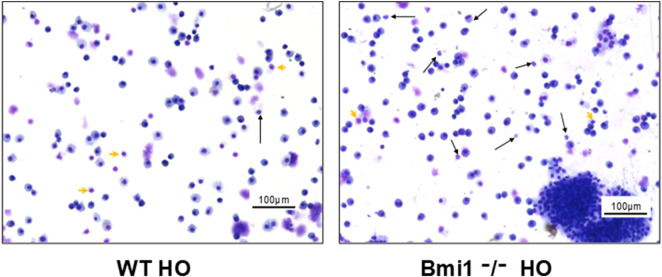
Analysis of Bronchoalveolar lavage fluid (BALF) from *Bmi1* ^−/−^ and *WT* mice exposed to hyperoxia. **(A)** *WT* mice, **(B)** *Bmi1* ^−/−^ mice. Representative Diff quick stained images from n = 6 mice per group. Neutrophils (black arrows) and individual lymphocytes (green arrowheads). Scale bar 100 μm.

There was a significant increase in polymorphonuclear cell migration and accumulation of lymphocytes and macrophages in *Bmi1* ^*−/−*^ mice versus *WT* mice exposed to hyperoxia (Figure 6).

### 3.7 *Bmi1* depletion was deleterious to lung function

A flexiVent analysis was performed for *WT* and *Bmi1* ^*−/−*^ mice after normoxia or hyperoxia exposure to determine if Bmi1 depletion was related to lung functionality in HALI.

**FIGURE 7:**
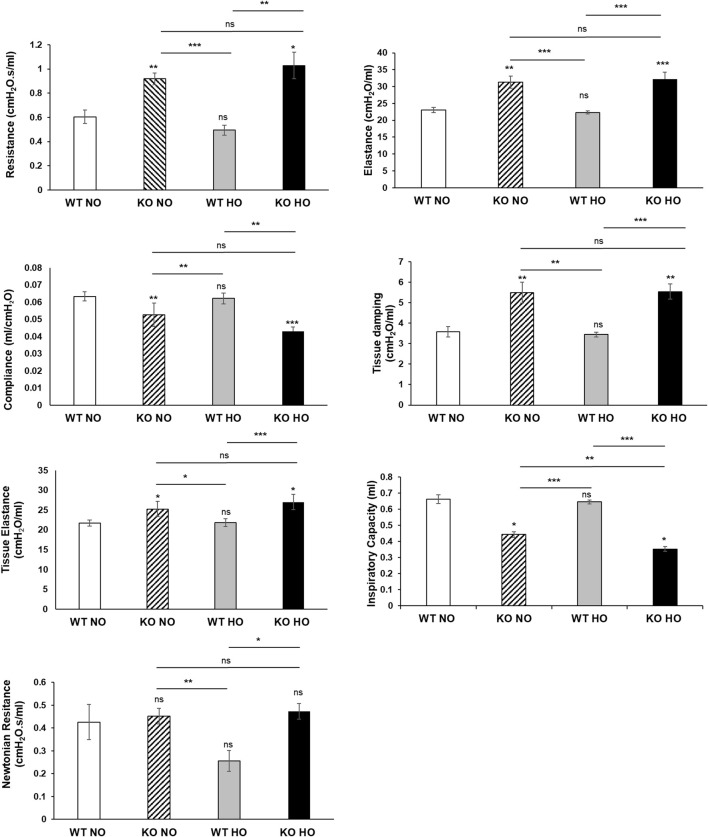
Analysis of lung function. *Bmi1* ^*−/−*^ and *WT* mice were exposed to normoxia and hyperoxia for 48 h. Lung functional analysis was carried out using flexiVent. Various lung parameters were evaluated. **(A)** Resistance **(B)** Elastance **(C)** Compliance **(D)** Tissue damping **(E)** Tissue elastance **(F)** Inspiratory capacity **(G)** Newtonian resistance. Data are represented as Mean ± SEM for n = 6 mice per group. Two-way ANOVA, * p-value ≤0.05, ** p-value ≤0.01, ***p-value≤0.001.

Functional analysis showed that *Bmi1* knockout vs. *WT* mice had decreased IC (Figure 7F) and Cst (Figures 7C,F). The decrease was more significant in *Bmi1* ^*−/−*^ mice exposed to hyperoxia. Ers (Figures 7B,E) and Rrs (Figure 7A) was increased in *KO* versus *WT* mice.

## 4 Discussion

At a molecular level, hyperoxia causes cell death, inflammation, mitochondrial dysfunction, and DNA fragmentation, whereas at a macro level it induces changes in lung structure and functionality (Kolliputi and Waxman, 2009a; Kolliputi and Waxman, 2009b; Kolliputi et al., 2010; Waxman and Kolliputi, 2009; Narala et al., 2018; Hernández-Cuervo H et al., 2022). Our group recently demonstrated how diminished expression of BMI1 in human lung epithelial cells is associated with mitochondrial alterations including increased production of ROS and significant decrease in OCR, basal respiratory capacity, proton leak and ATP production (Hernández-Cuervo H et al., 2022). Decreased BMI1 expression is related to poor cell survival and dysregulation of the cell cycle (Bhattacharya et al., 2015; Banerjee Mustafi et al., 2017; Banerjee Mustafi et al., 2016). For the first time, an *in vivo* investigation established a link between BMI1 and ALI, advancing the understanding of this nosological entity that is still poorly understood.

*Bmi1* depletion was positively correlated with decreased expression of Drp1 (Table 1). Kosmider and colleagues (2019) found impaired mitochondrial dysfunction associated with decreased DRP1 protein in human epithelial cells in pulmonary emphysema. Although a balance is needed between mitochondrial fusion and fission, a decrease of DRP1 in Type II alveolar epithelial cells (AECII) contributes to cell death (Kosmider et al., 2019). This study did not investigate the subjacent mechanism by which mouse lung cells are affected downstream of the Drp1 decrease. Still, the relationship between *Bmi1* genetic deletion and decreased Drp1 levels is evident, and can be explained, in part, by the mitochondrial alterations observed in our model. It is important to emphasize that changes in protein expression may differ between cell line or primary cell cultures (*in vitro* model) and proteins obtained from tissues (*in vivo* model) due to the multicellularity and complexity of the latter (Lacroix et al., 2018).

Pten tumor suppressor protein levels were significantly increased in *Bmi1* ^−/−^ mice, as found in BMI1, silenced H441 cells (Hernández-Cuervo H et al., 2022), indicating that PTEN protein expression is inversely regulated by BMI1 expression (Hernández-Cuervo H et al., 2022; Fan et al., 2009; Yang et al., 2019). The downstream Pten activation in lung tissue is in accordance with human lung epithelial cell data (Hernández-Cuervo H et al., 2022). The expression of Pten is enhanced by the suppression of Bmi1, which in turn triggers an increase in pAKT (Ser 473) protein signaling. High levels of pAKT could inhibit PGC-1α as reported in our recent *in vitro* model, partially explaining the changes in mitochondrial metabolism (Li et al., 2007; Rodgers et al., 2010). The lack of Bmi1 expression in combination with an increase in Pten and pAkt levels inhibits the Akt/PKB pathway that interferes with the cell cycle and turnover, confirming the crucial role of BMI1 in cell survival (Fan et al., 2009; Gupta et al., 2017; Nacerddine et al., 2012).

Determination of the oxygen consumption rate in live mitochondria is a challenge because of the gap between organ collection and mitochondria isolation (up to 6 h after euthanasia). This fact could be related to the alteration in the OCR parameters evaluated. However, the same method at the same time was used to isolate the live mitochondria in all the groups of mice studied (*WT* and *KO* normoxia and hyperoxia exposed). Our research showed the same trend observed previously in human lung epithelial cells and human lung isolated live mitochondria (Hernández-Cuervo et al., 2022). We observed a decrease in the respiratory rate of the mitochondria isolated from the *Bmi1* ^*−/−*^ and *WT* hyperoxia mice. These changes were associated with a decrease in ATP production and spare respiratory capacity; similar findings were described by Das (2013) for mouse lung epithelial cells (Figure 2) (Das, 2013). Bleomycin-injured AECII exhibited a decreased OCR that worsened with increased bleomycin (Shaghaghi et al., 2021). In this *in vivo* model, hyperoxia injury induced a decreased OCR in lung mitochondria. The decreased OCR in *Bmi1* ^*−/−*^ mice, even under normoxia, provides further evidence that Bmi1 protein expression is necessary for proper mitochondrial respiration in basal conditions. Additionally, a decrease in Bmi1 expression and increase in ROS due to hyperoxia worsens the mitochondrial response in *Bmi1* ^*−/−*^ mice (Kellner et al., 2017; Hernández-Cuervo et al., 2022; Herrero et al., 2018b; Chacinska et al., 2009).

ALIS was used to establish the level of damage in lung architecture, which is directly related to the severity of the injury (Sue et al., 2004; Jain et al., 2008). The ALIS corroborated our hypothesis that decreased Bmi1 expression worsens HALI (Figures 3, 4; Table 2). Hallmarks of ALI/ARDS include migration of PMN blood cells into alveolar spaces, cytokine storm, alveolar-capillary barrier dysfunction with an increase in lung permeability, mitochondrial damage, and increased oxidative stress (Matute-Bello et al., 2011; Zaher et al., 2007; Martin and Matute-Bello, 2011). This study showed that *Bmi1* ^*−/−*^ mice exposed to hyperoxia had a very severe disease state with increased alveolar wall thickness, hemorrhage, and disruption of the alveolar-capillary barrier leading to increased lung permeability. Blood cell migration and increased lung permeability are additional characteristics of ALI (Bhargava and Wendt, 2012; Grommes and Soehnlein, 2011). BALF protein analysis, cellularity and EBD extravasation show the highest values in *Bmi1* ^*−/−*^ exposed to HO with evidence of increased infiltration of immune cells (PMN, lymphocytes, and macrophages) and pulmonary edema (Figures 5, 6).

Mice with different genetic backgrounds were studied to assess the molecular basis of HALI (Fukumoto et al., 2016; Narala et al., 2018; Cox et al., 2015; Sidramagowda Patil et al., 2021). This research approach indicated several risks and protective factors for HALI, and the disease has been examined, not only with molecular markers, but with a direct evaluation of lung injury (histology) and function (flexiVent). Spirometry is the most commonly performed lung function test in humans. In animal studies, there exists a similar way to evaluate lung functionality. FlexiVent is the gold standard technique to assess lung function *in vivo* models with high reproducibility and efficacy. Data from lung function analysis *in vivo* showed that adequate Bmi1 expression is necessary to maintain lung compliance and the ability of lung tissue to expand and stretch as needed (it is closely related to lung elastance). *Bmi1* deletion was associated with the loss of compliance and limited inspiratory capacity, an increase in lung resistance and worsening symptoms, as present in subjects with ALI/ARDS. These results showed that the four features of lung function (compliance, inspiratory capacity, resistance, and elastance) are drastically affected in *Bmi1* ^*−/−*^ mice exposed to hyperoxia versus *WT* mice or *Bmi1* ^*−/−*^ mice under normoxic conditions, confirming that Bmi1 loss has a deleterious effect on the response to hyperoxia injury (Figure 7) (Verjans et al., 2018). These findings demonstrate that Bmi1 expression is required for normal lung function and that hyperoxia, in addition to the lack of Bmi1, exacerbates pulmonary dysfunction (Figure 7).

In conclusion, this study demonstrates that decreased Bmi1 expression exacerbates HALI in mice. The deletion of *Bmi1* was related to severe onset of HALI, a decrease in lung function parameters, an increase in lung permeability, and cellular and protein extravasation. These results establish the importance of Bmi1 in the response to injury caused by hyperoxia and establishes BMI1 as a molecular candidate in HALI. Future studies will evaluate if Bmi1 overexpression can repair ALI caused by *Bmi1* deletion and oxidant exposure. The role of BMI1 in subjects also should be evaluated to determine if BMI1 is a therapeutic target to manage ARDS.

This study is limited by the absence of direct measurements of mitochondrial membrane potential (ΔΨm) and ROS levels, and by the potential for partial mitochondrial degradation during the 6-h post-euthanasia processing period. Because all groups underwent identical handling, these factors should not have introduced systematic bias. Future work will apply live-cell imaging and quantitative mitophagy assays (PINK1/Parkin pathway) to validate the mechanistic insights proposed here (Wasilewski et al., 2017).

## Data Availability

The original contributions presented in the study are included in the article/Supplementary Material, further inquiries can be directed to the corresponding author.

## Supplementary material

SUPPLEMENTARY FIGURE S1 Phenotypical representation of the mouse model. **(A)** Photograph from *WT* and *Bmi1* ^−/−^ mice. **(B)** Bodyweight for wild-type (n = 83) and Bmi1 knockout (n = 83) mice, two ways ANOVA, *** p-value ≤0.001. **(C)** Relative fold change of *Bmi1* transcript normalized to β-Actin, n = 3 (Mean ± SEM), two ways ANOVA, ns = not significant, ** p-value ≤0.01 versus wild-type controls.
