# Supplementary figures and images for: Bmi1 deficiency exacerbates hyperoxia-induced acute lung injury in mice
Source: Front Physiol. 2025 Nov 13;16:1695456. doi: 10.3389/fphys.2025.1695456 (PMC12658778; doi:10.3389/fphys.2025.1695456)

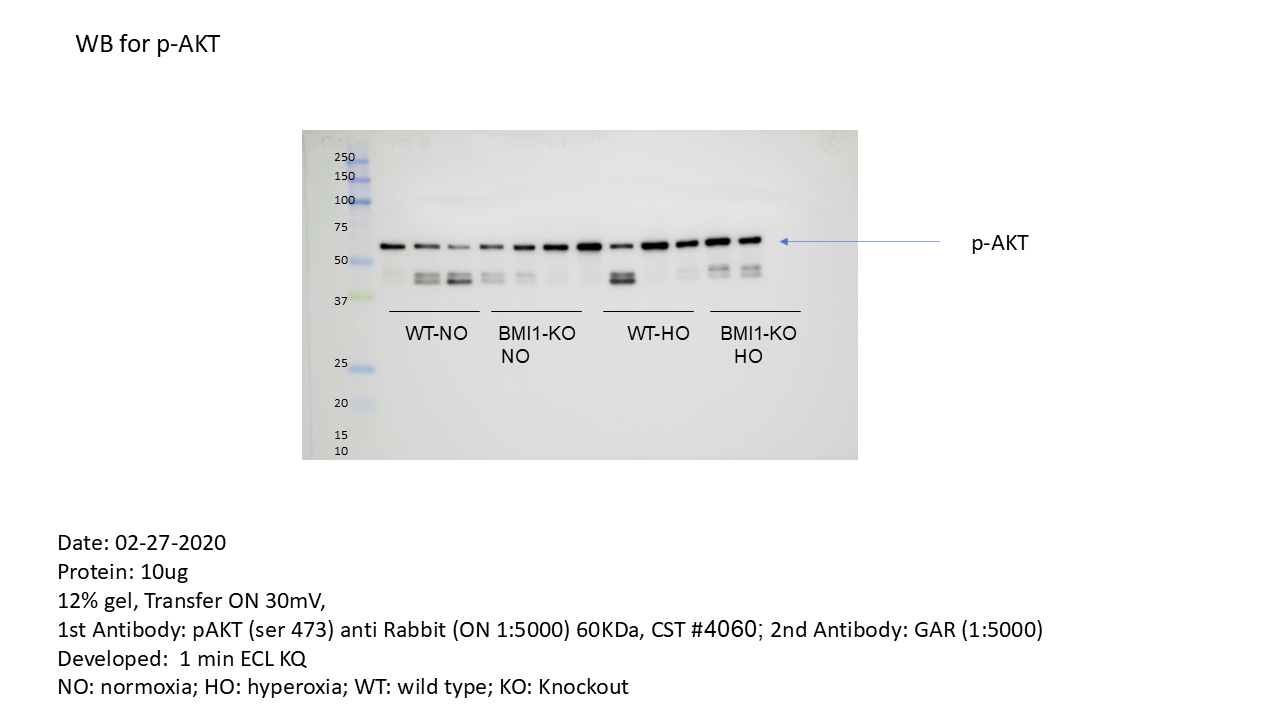

Supplement: Supplementary file 1 [file Image6.tif]

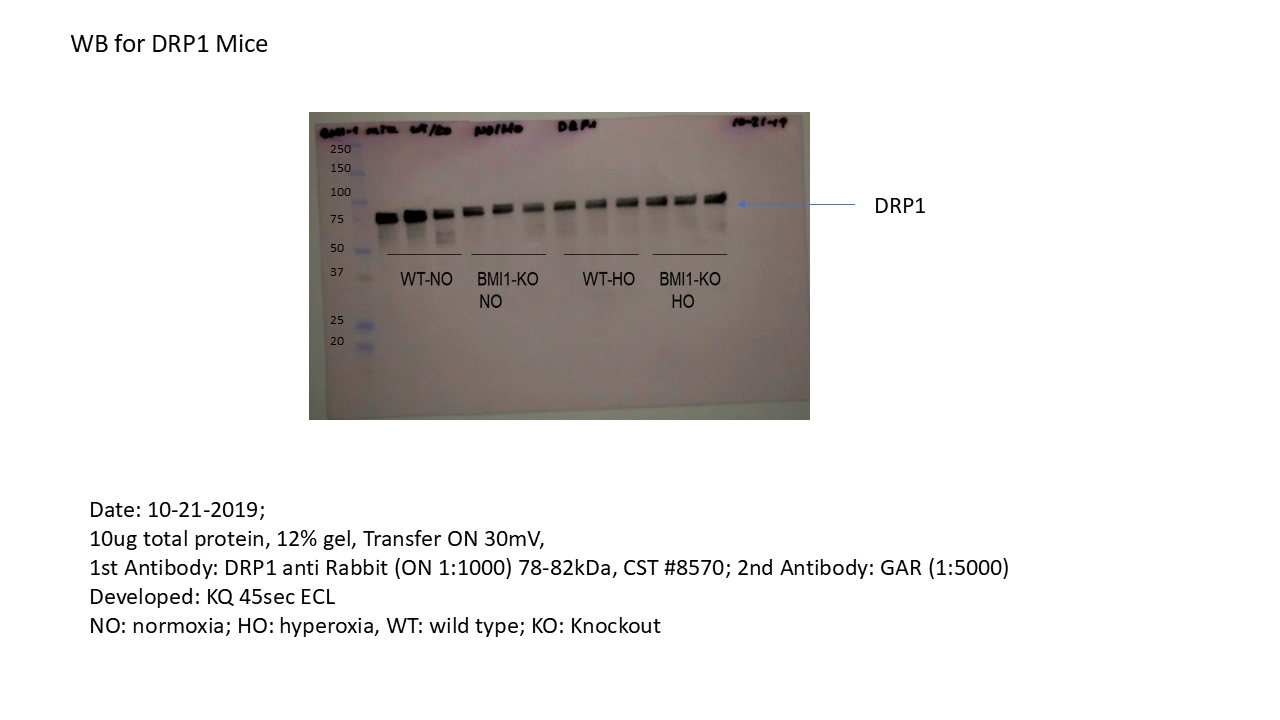

Supplement: Supplementary file 4 [file Image3.tif]

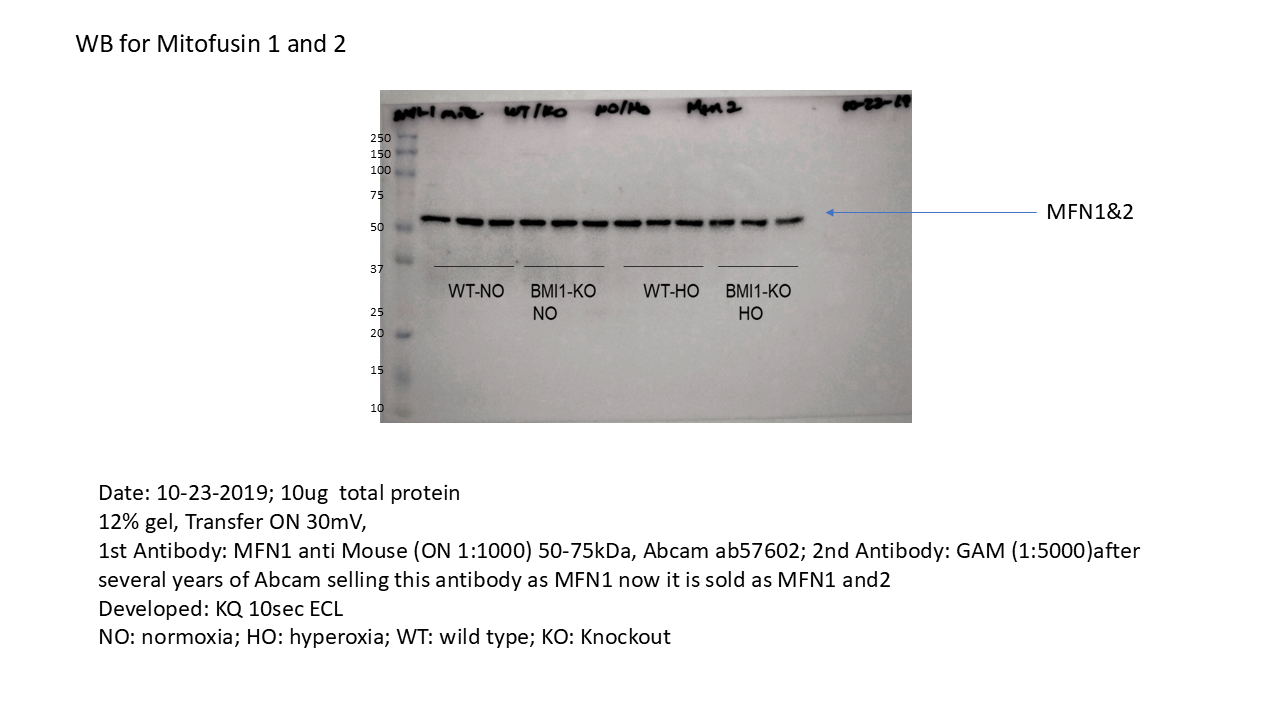

Supplement: Supplementary file 5 [file Image4.tif]

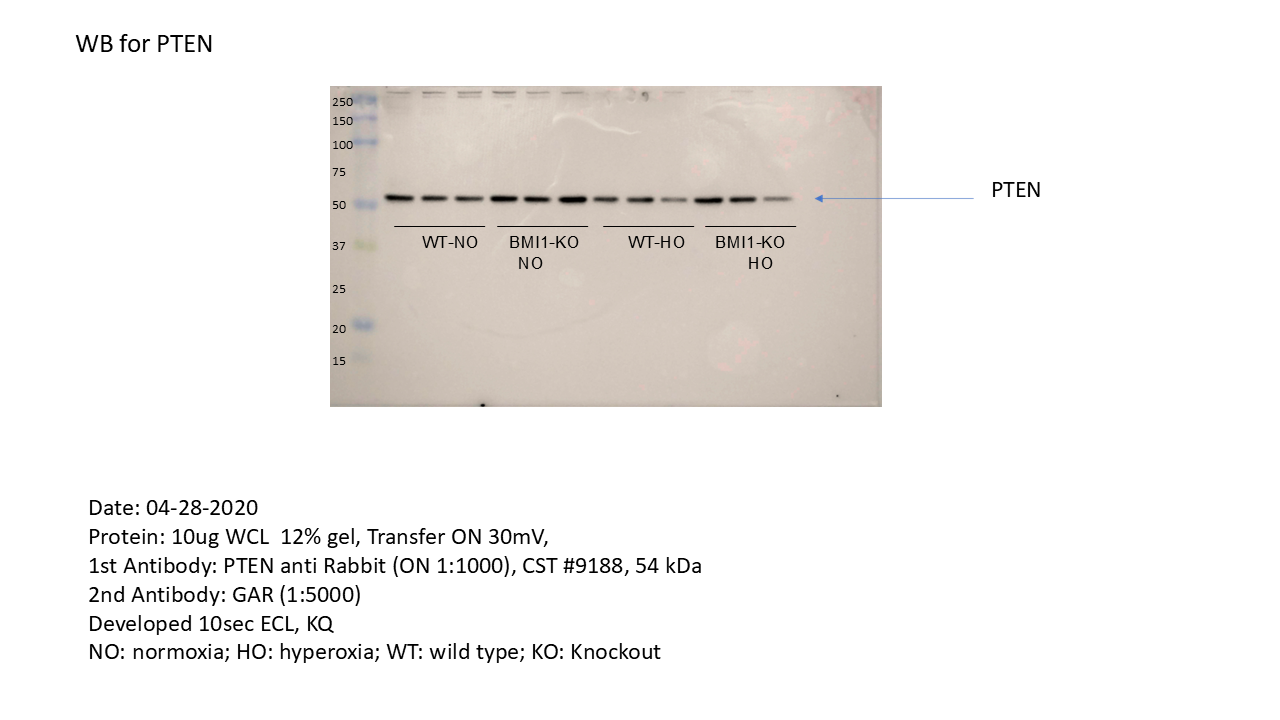

Supplement: Supplementary file 6 [file Image9.tif]

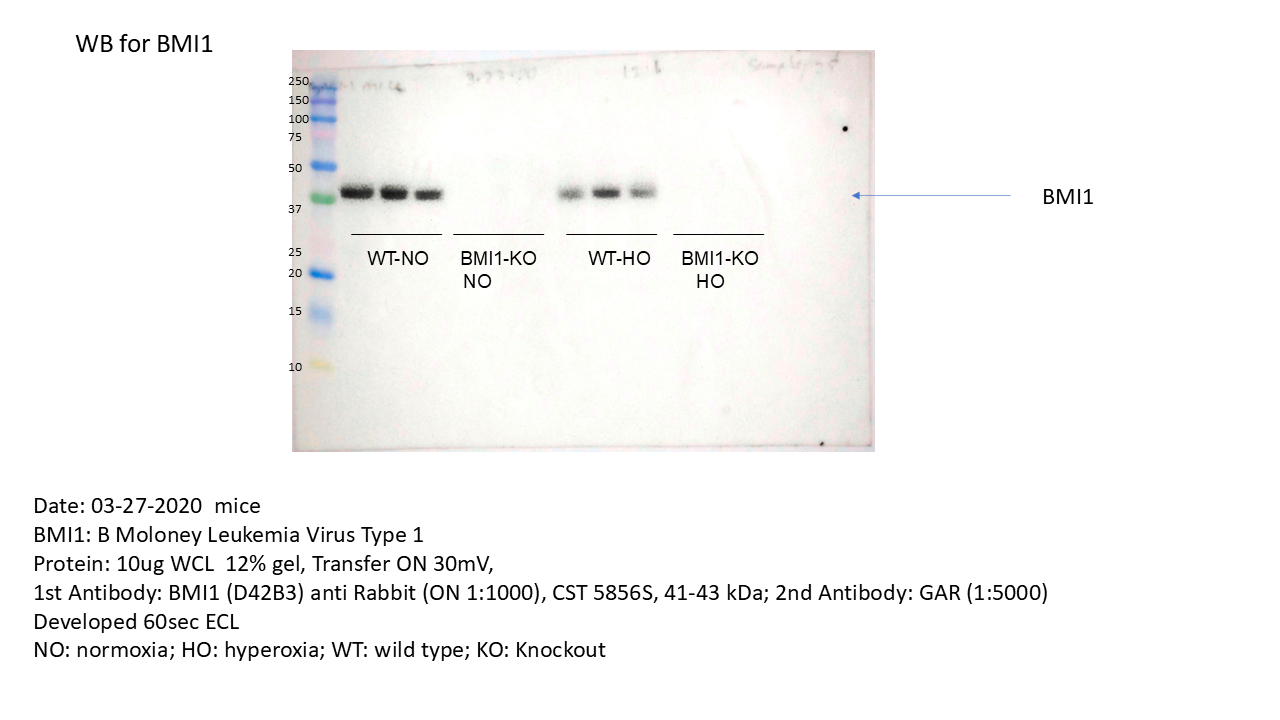

Supplement: Supplementary file 7 [file Image2.tif]

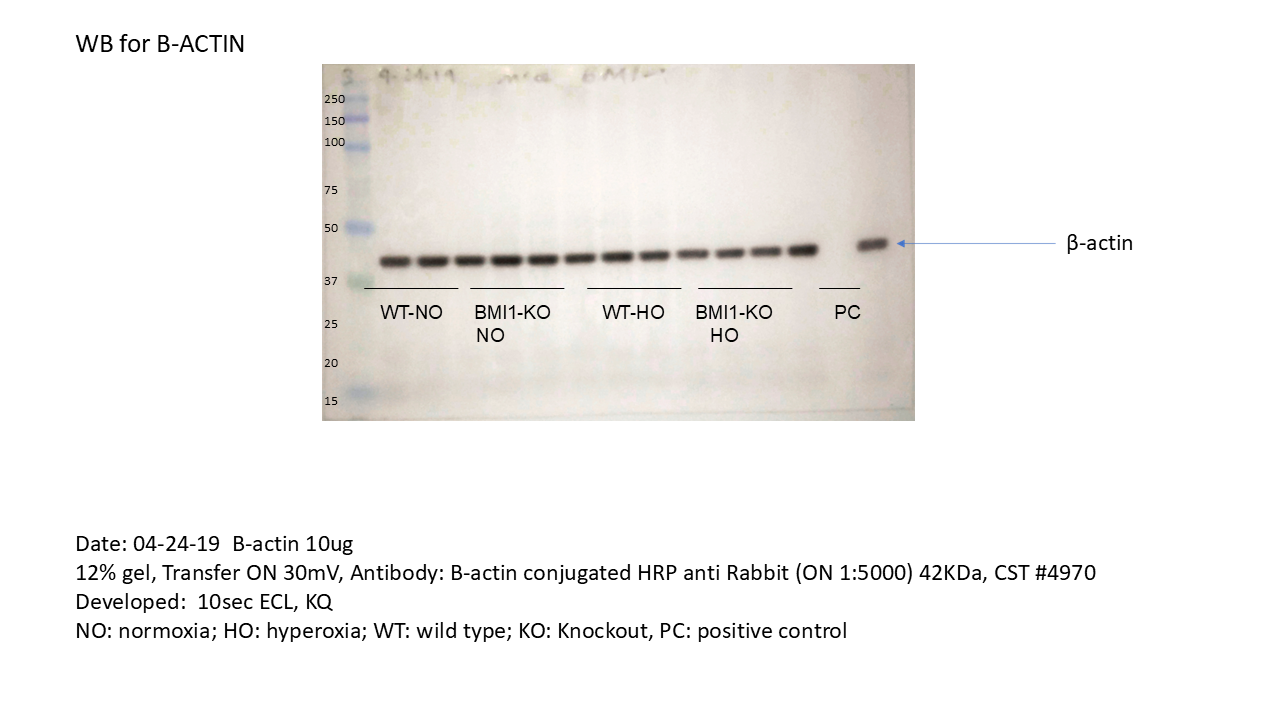

Supplement: Supplementary file 8 [file Image11.tif]

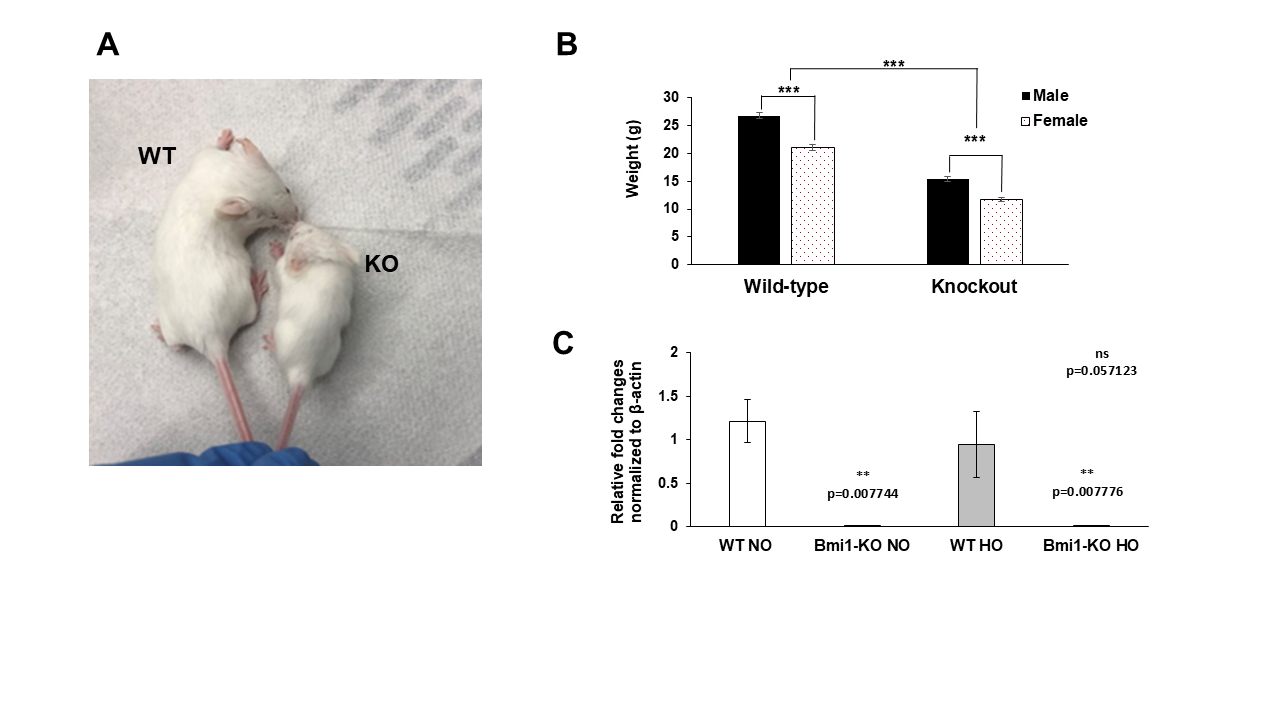

Supplement: Supplementary file 9 [file Image1.tif]

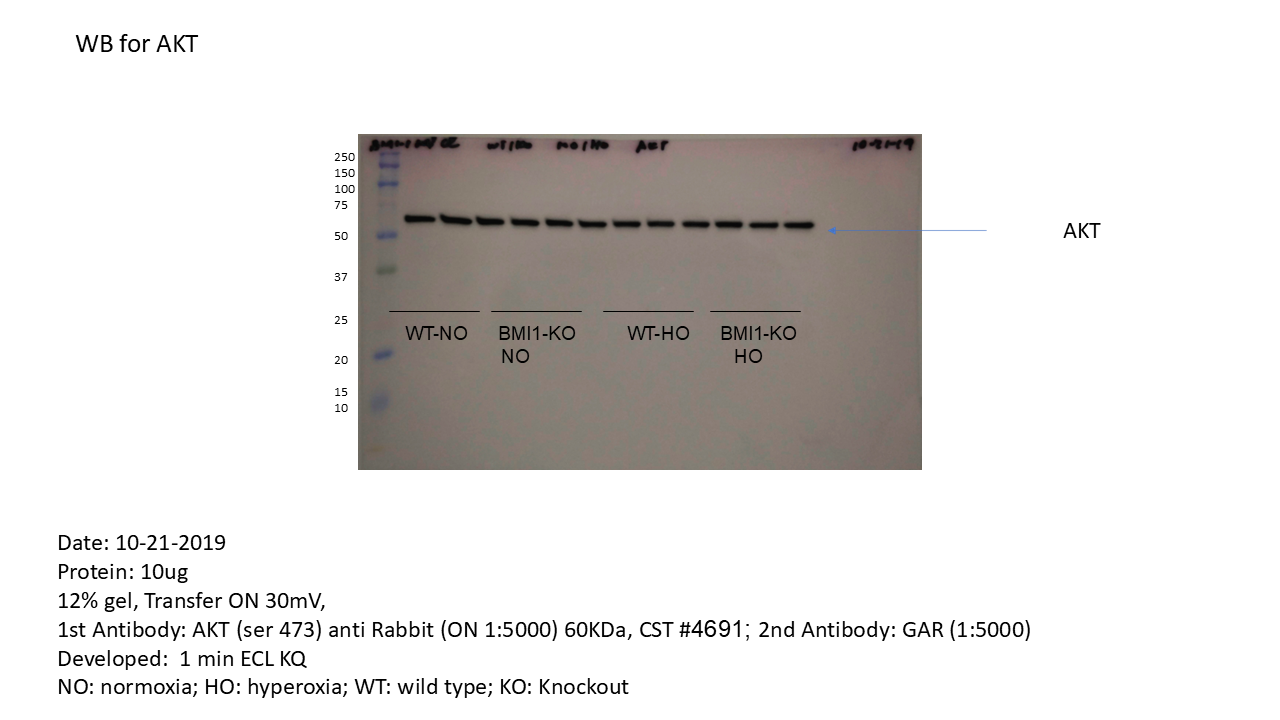

Supplement: Supplementary file 10 [file Image10.tif]

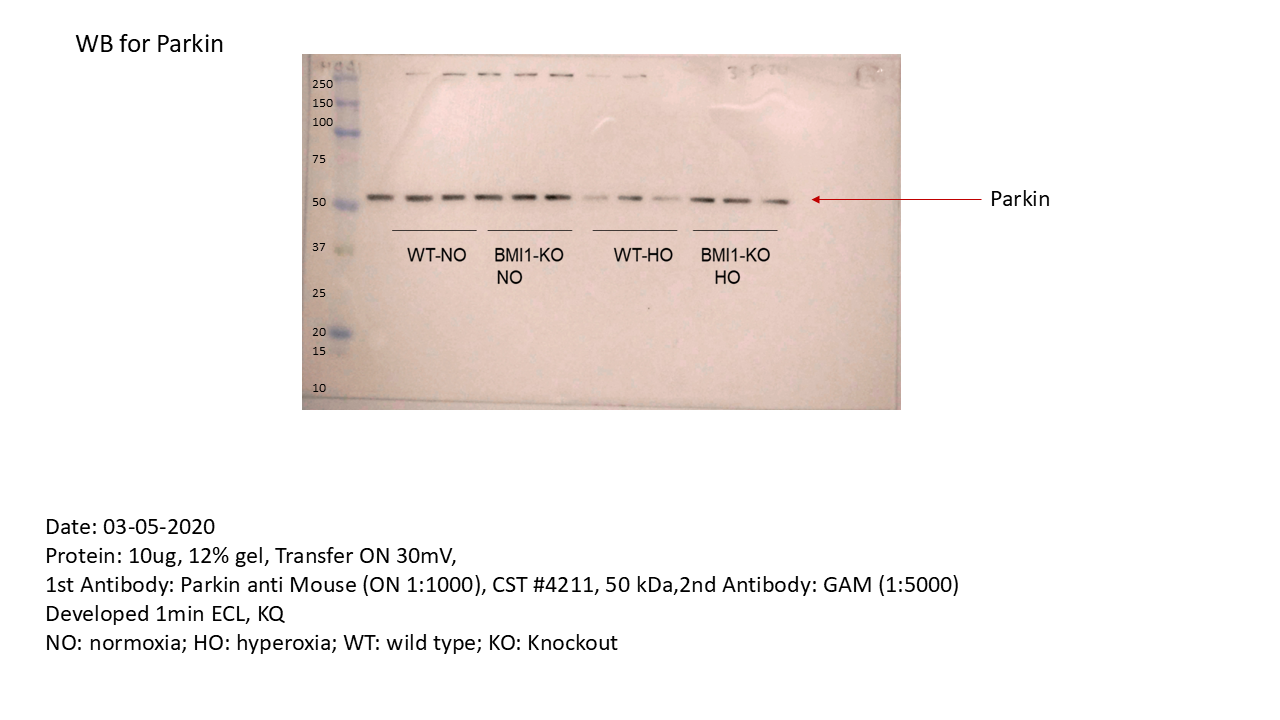

Supplement: Supplementary file 11 [file Image7.tif]

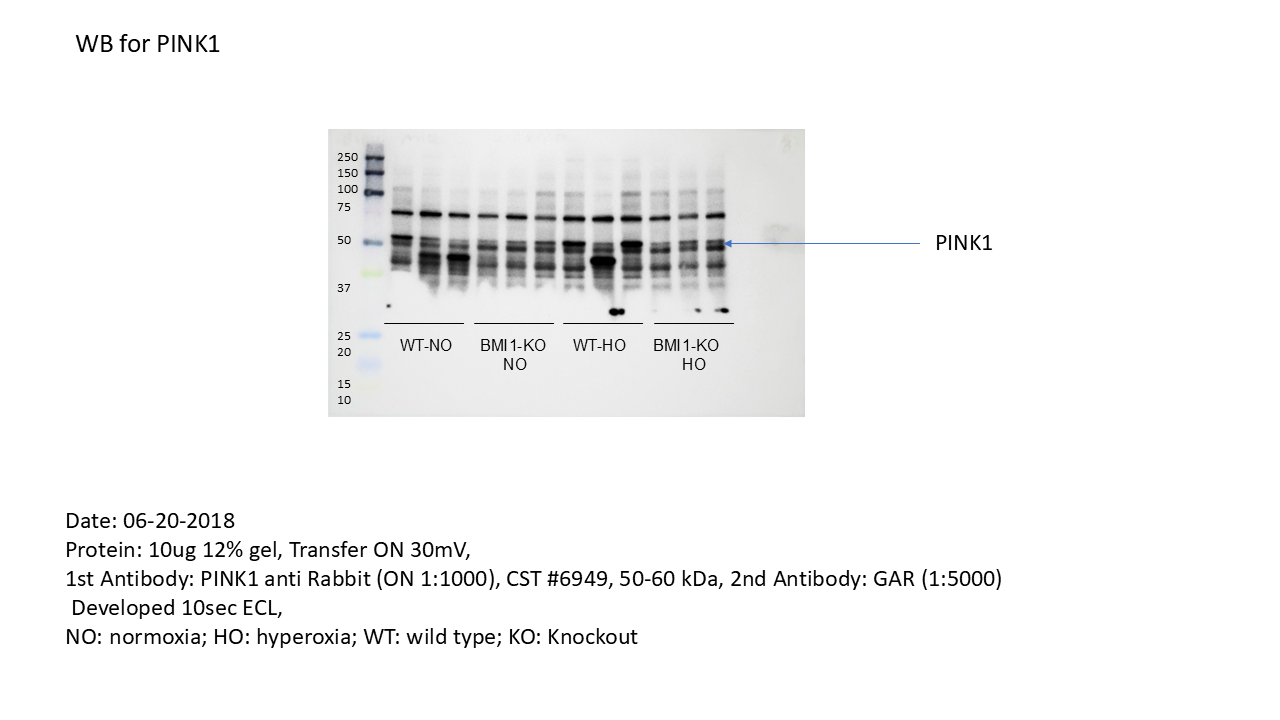

Supplement: Supplementary file 13 [file Image8.tif]

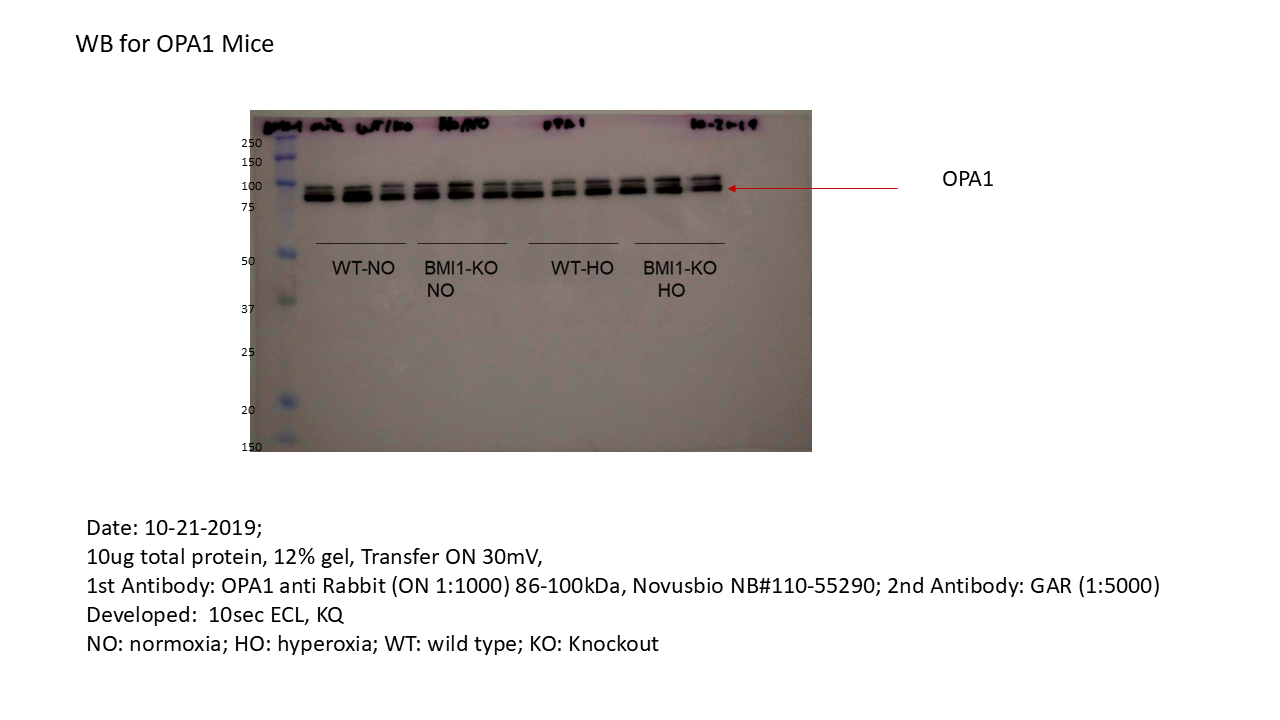

Supplement: Supplementary file 14 [file Image5.tif]
