# Supplementary material for: Bmi1 deficiency exacerbates hyperoxia-induced acute lung injury in mice
Source: Front Physiol. 2025 Nov 13;16:1695456. doi: 10.3389/fphys.2025.1695456 (PMC12658778; doi:10.3389/fphys.2025.1695456)
Supplement: Supplementary file 2 [file Table1.docx]

**Supplement Table 1. Antibodies and conditions of western blot**

| **Antibody Name** | **Primary**  **Concentration** | **Solution** | **Incubation** | **MW (kDa)** | **Secondary** | **Secondary**  **Concentration** | **Company** | **Catalog**  **Number** |
| --- | --- | --- | --- | --- | --- | --- | --- | --- |
| **AKT** | 1:1000 | 5% BSA | ON | 60 | Goat anti Rb | 1:5000 | CST | #4691 |
| **p-AKT (Ser 473)** | 1:1000 | 5% BSA | ON | 60 | Goat anti Rb | 1:5000 | CST | #4060 |
| **β-Actin** | 1:5000 | 5% BSA | 45 min | 45 | Conjugated |  | CST | #12262 |
| **BMI1** | 1:1000 | 5% BSA | ON | 41-43 | Goat anti Rb | 1:5000 | CST | #5856 |
| **DRP1** | 1:1000 | 5% BSA | ON | 78-82 | Goat anti Rb | 1:5000 | CST | #8570 |
| **Mitofusin 1** | 1:1000 | 5% BSA | ON | 84 | Goat anti Ms | 1:5000 | Abcam | ab 57602 |
| **OPA1** | 1:1000 | 5% BSA | ON | 86-111 | Goat anti Rb | 1:5000 | Novusbio | NB110-55290 |
| **Parkin** | 1:1000 | 5% BSA | ON | 50 | Goat anti Ms | 1:5000 | CST | #4211 |
| **PINK1** | 1:1000 | 5% BSA | ON | 50-60 | Goat anti Rb | 1:5000 | CST | #6949 |
| **PTEN** | 1:1000 | 5% BSA | ON | 54 | Goat anti Rb | 1:5000 | CST | #9188 |

ON: Overnight. CST: Cell Signaling Technologies
