# Supplementary material for: Bmi1 deficiency exacerbates hyperoxia-induced acute lung injury in mice
Source: Front Physiol. 2025 Nov 13;16:1695456. doi: 10.3389/fphys.2025.1695456 (PMC12658778; doi:10.3389/fphys.2025.1695456)
Supplement: Supplementary file 3 [file Presentation1.pptx]

## Slide 1
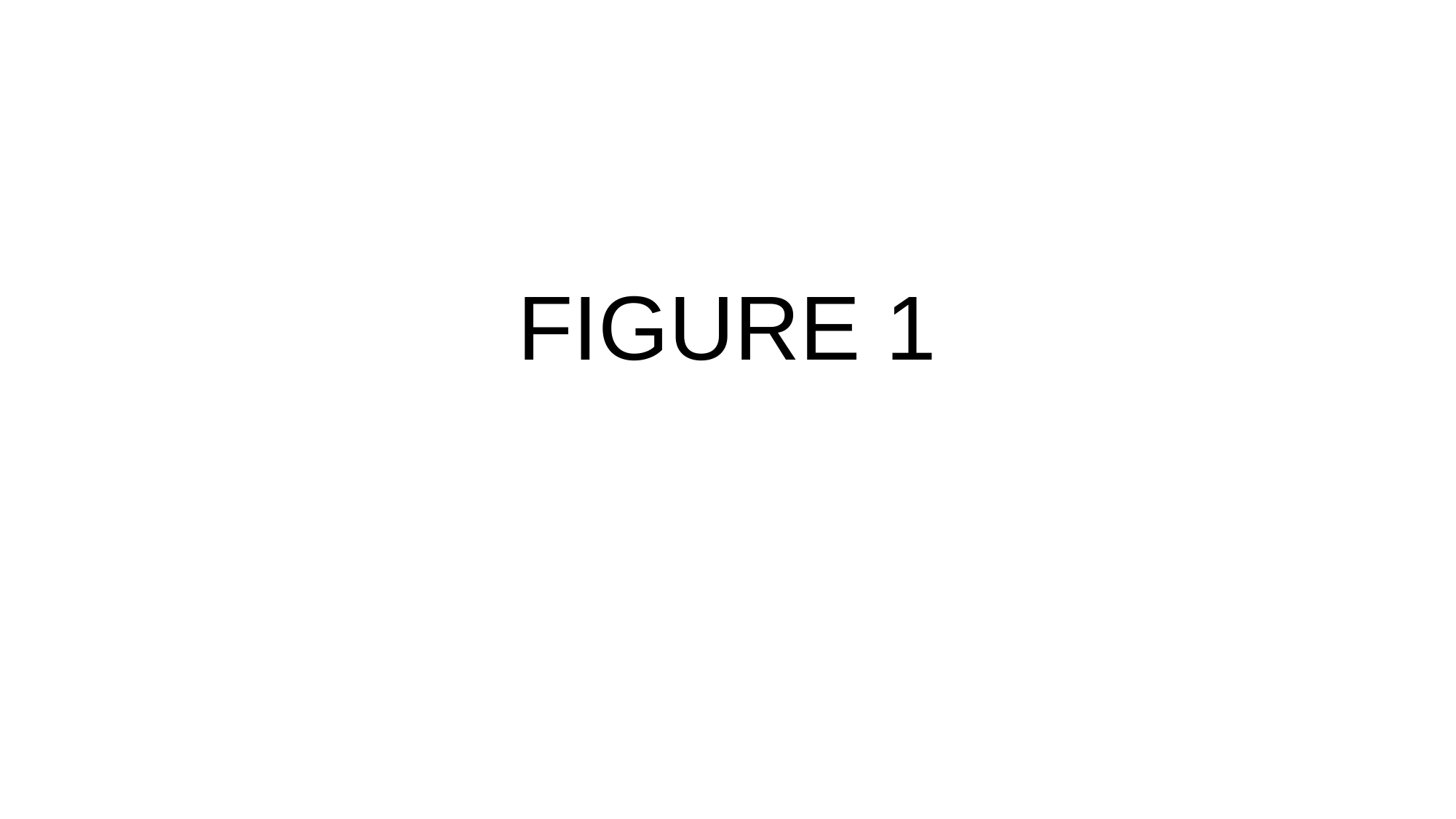

# FIGURE 1

## Slide 2
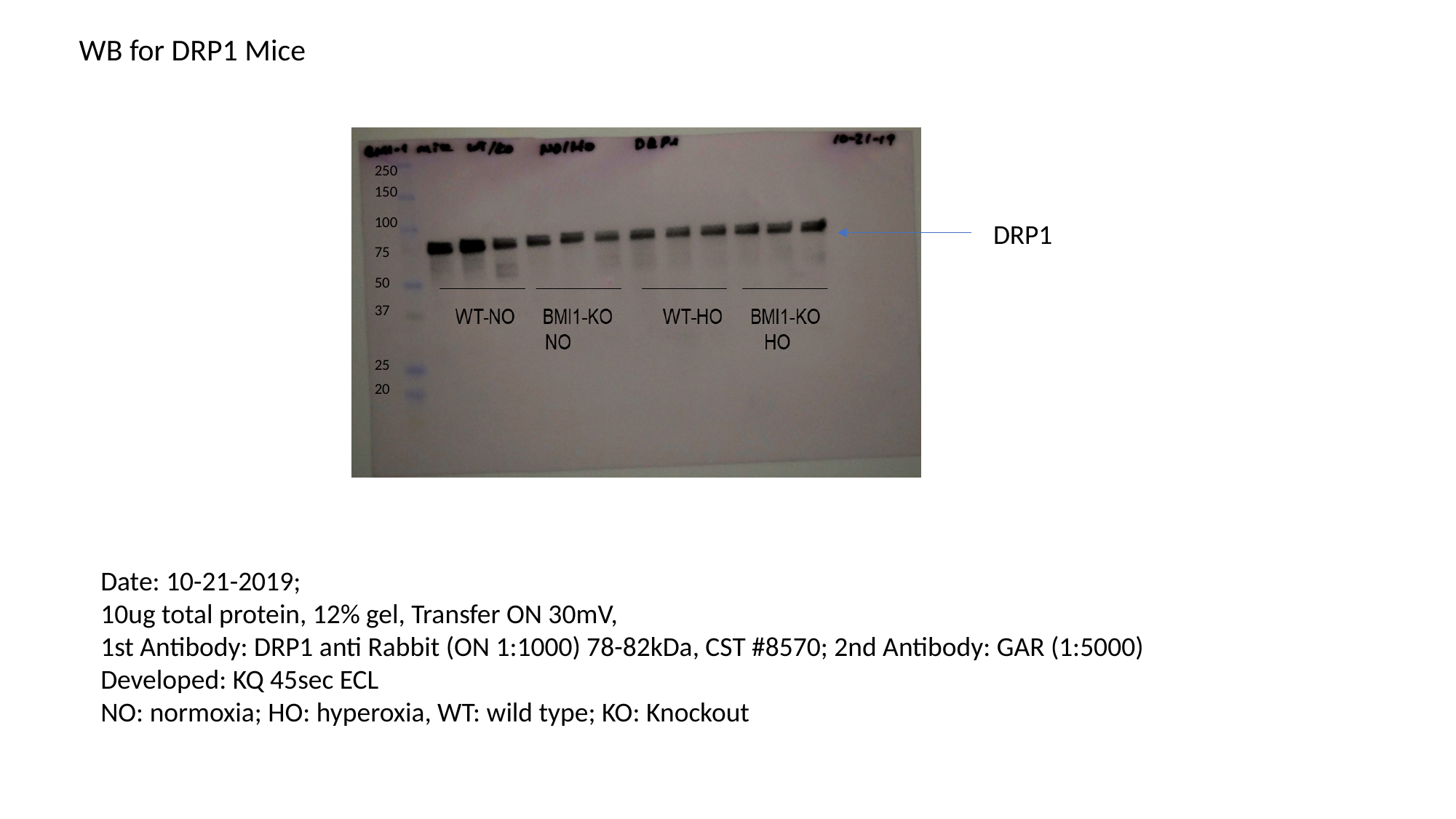

WB for DRP1 Mice
250
150
100
75
50
37
25
20
DRP1
Date: 10-21-2019;
10ug total protein, 12% gel, Transfer ON 30mV,
1st Antibody: DRP1 anti Rabbit (ON 1:1000) 78-82kDa, CST #8570; 2nd Antibody: GAR (1:5000)
Developed: KQ 45sec ECL
NO: normoxia; HO: hyperoxia, WT: wild type; KO: Knockout

## Slide 3
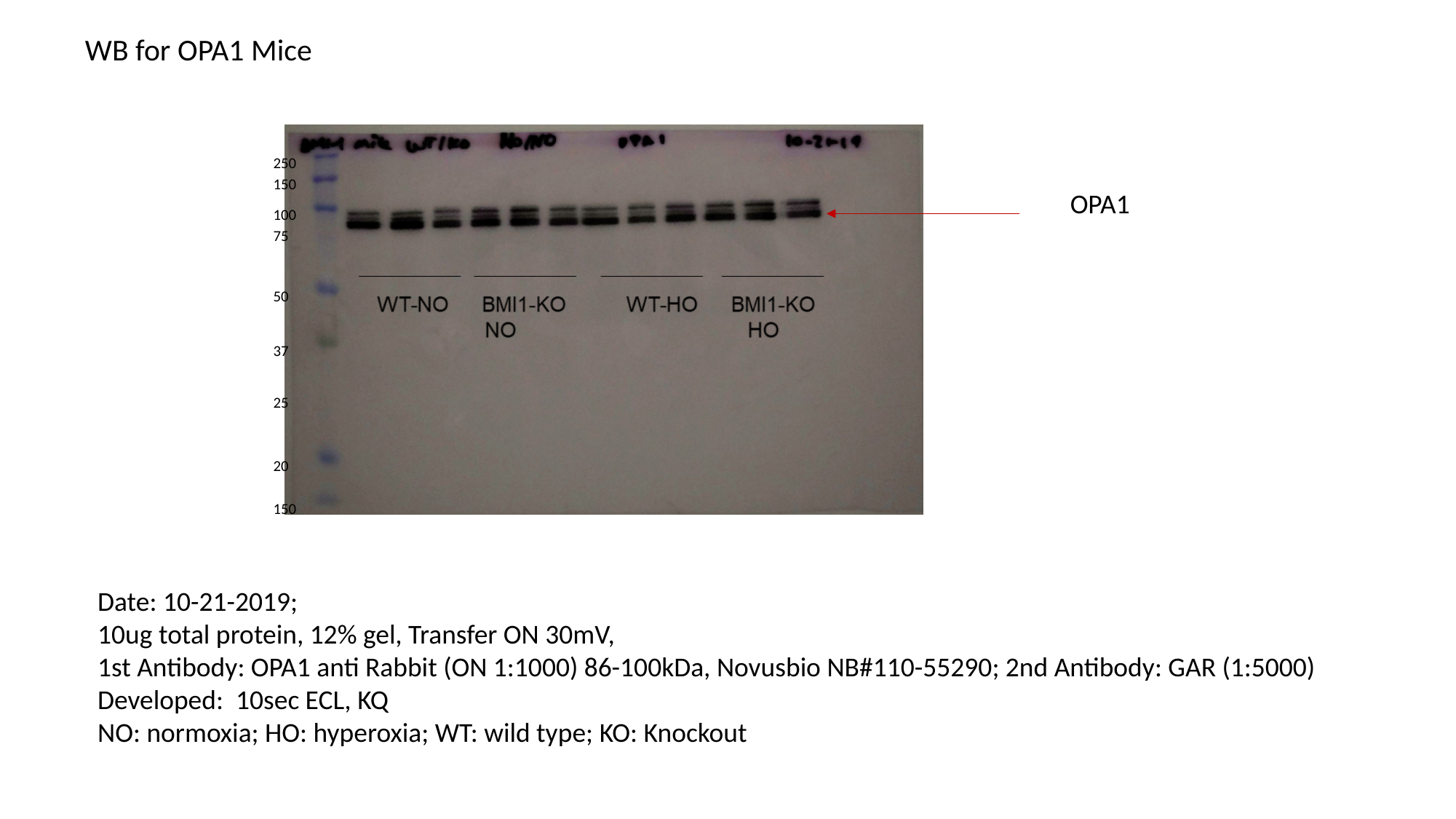

WB for OPA1 Mice
250
150
100
75
50
37
25
20
150
OPA1
Date: 10-21-2019;
10ug total protein, 12% gel, Transfer ON 30mV,
1st Antibody: OPA1 anti Rabbit (ON 1:1000) 86-100kDa, Novusbio NB#110-55290; 2nd Antibody: GAR (1:5000)
Developed: 10sec ECL, KQ
NO: normoxia; HO: hyperoxia; WT: wild type; KO: Knockout

## Slide 4
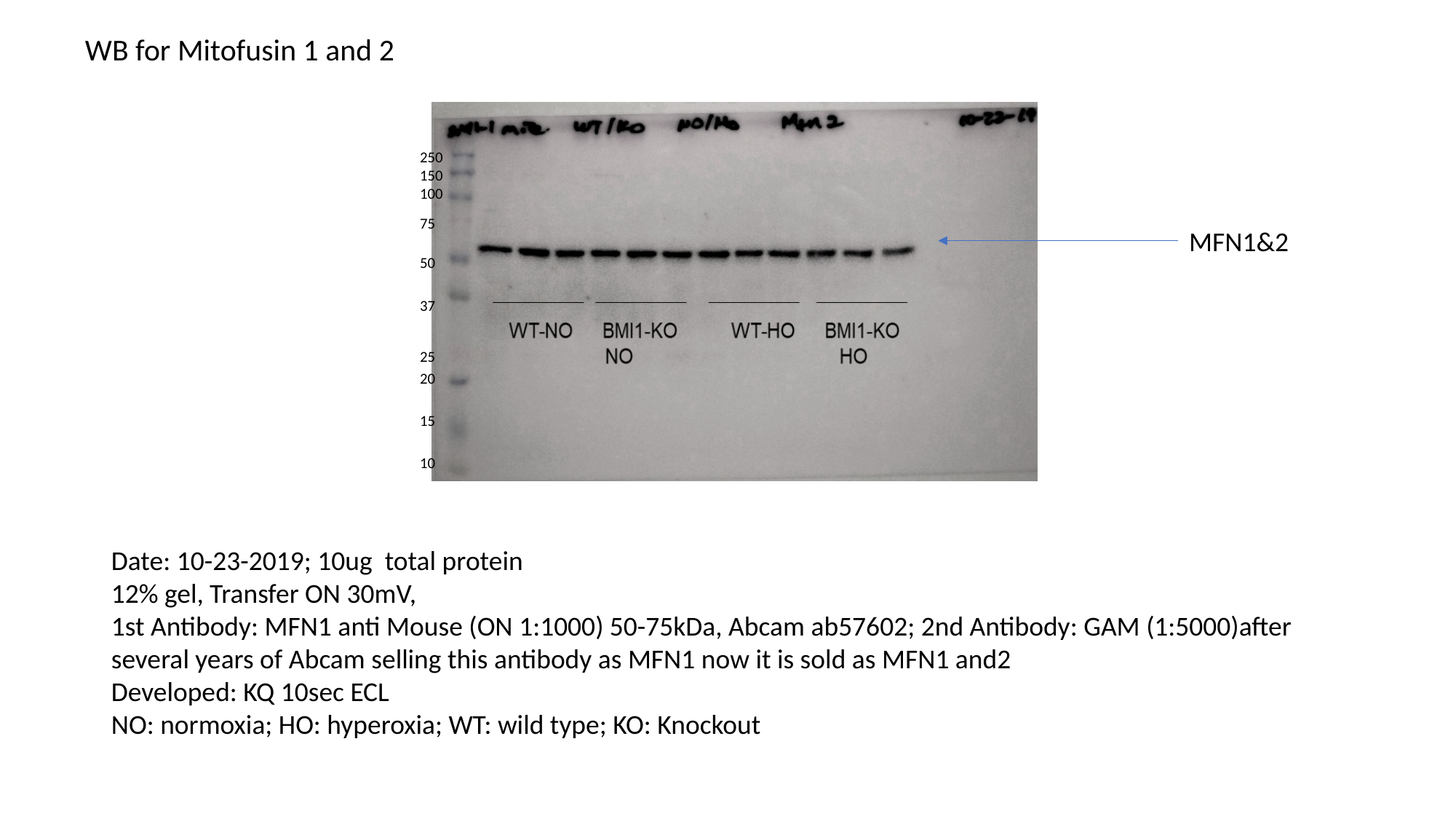

WB for Mitofusin 1 and 2
250
150
100
75
50
37
25
20
15
10
MFN1&2
Date: 10-23-2019; 10ug total protein
12% gel, Transfer ON 30mV,
1st Antibody: MFN1 anti Mouse (ON 1:1000) 50-75kDa, Abcam ab57602; 2nd Antibody: GAM (1:5000)after several years of Abcam selling this antibody as MFN1 now it is sold as MFN1 and2
Developed: KQ 10sec ECL
NO: normoxia; HO: hyperoxia; WT: wild type; KO: Knockout

## Slide 5
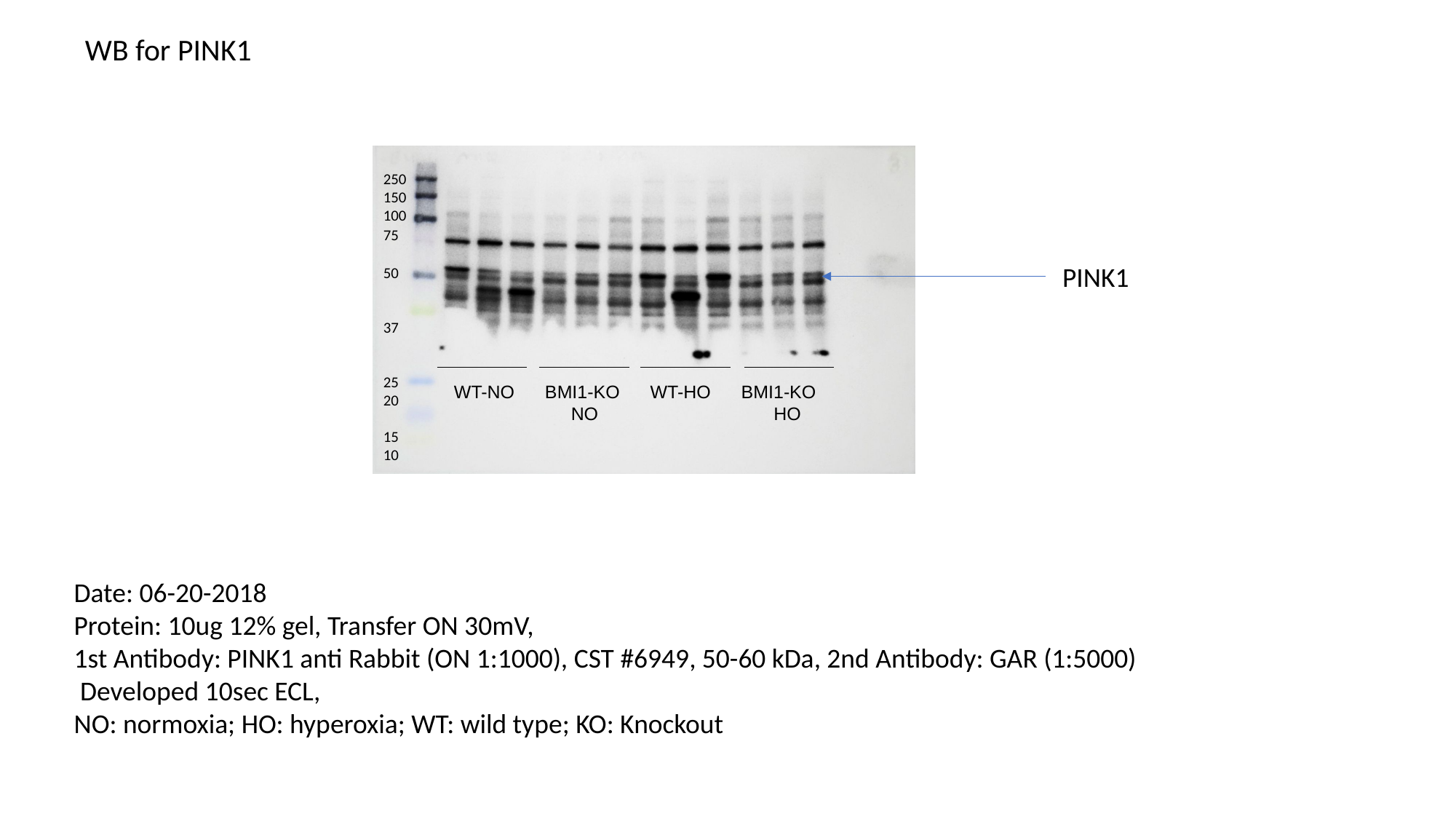

WB for PINK1
250
150
100
75
50
37
25
20
15
10
PINK1
WT-NO BMI1-KO WT-HO BMI1-KO
 NO 	 HO
Date: 06-20-2018
Protein: 10ug 12% gel, Transfer ON 30mV,
1st Antibody: PINK1 anti Rabbit (ON 1:1000), CST #6949, 50-60 kDa, 2nd Antibody: GAR (1:5000)
 Developed 10sec ECL,
NO: normoxia; HO: hyperoxia; WT: wild type; KO: Knockout

## Slide 6
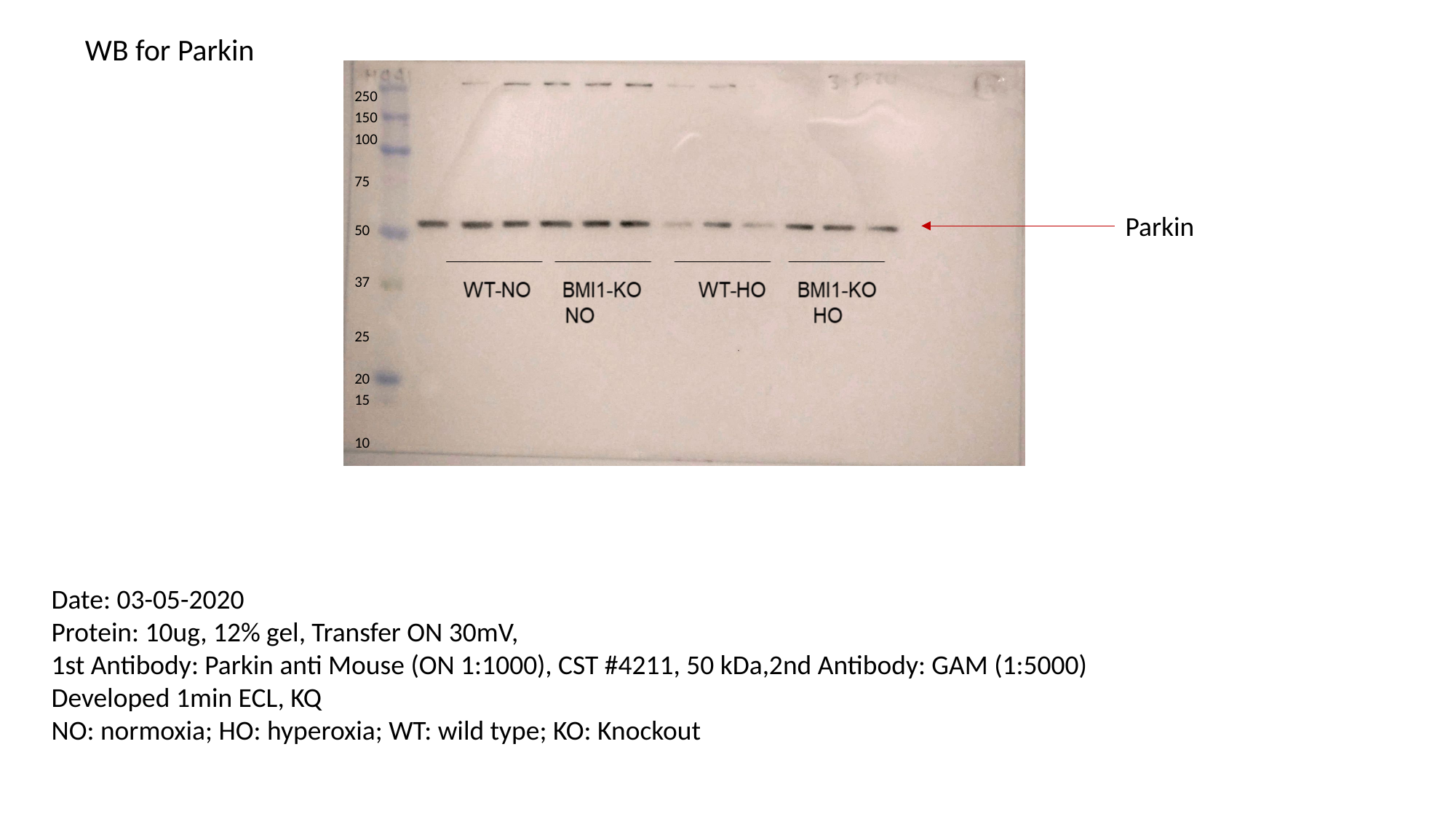

WB for Parkin
250
150
100
75
50
37
25
20
15
10
Parkin
Date: 03-05-2020
Protein: 10ug, 12% gel, Transfer ON 30mV,
1st Antibody: Parkin anti Mouse (ON 1:1000), CST #4211, 50 kDa,2nd Antibody: GAM (1:5000)
Developed 1min ECL, KQ
NO: normoxia; HO: hyperoxia; WT: wild type; KO: Knockout

## Slide 7
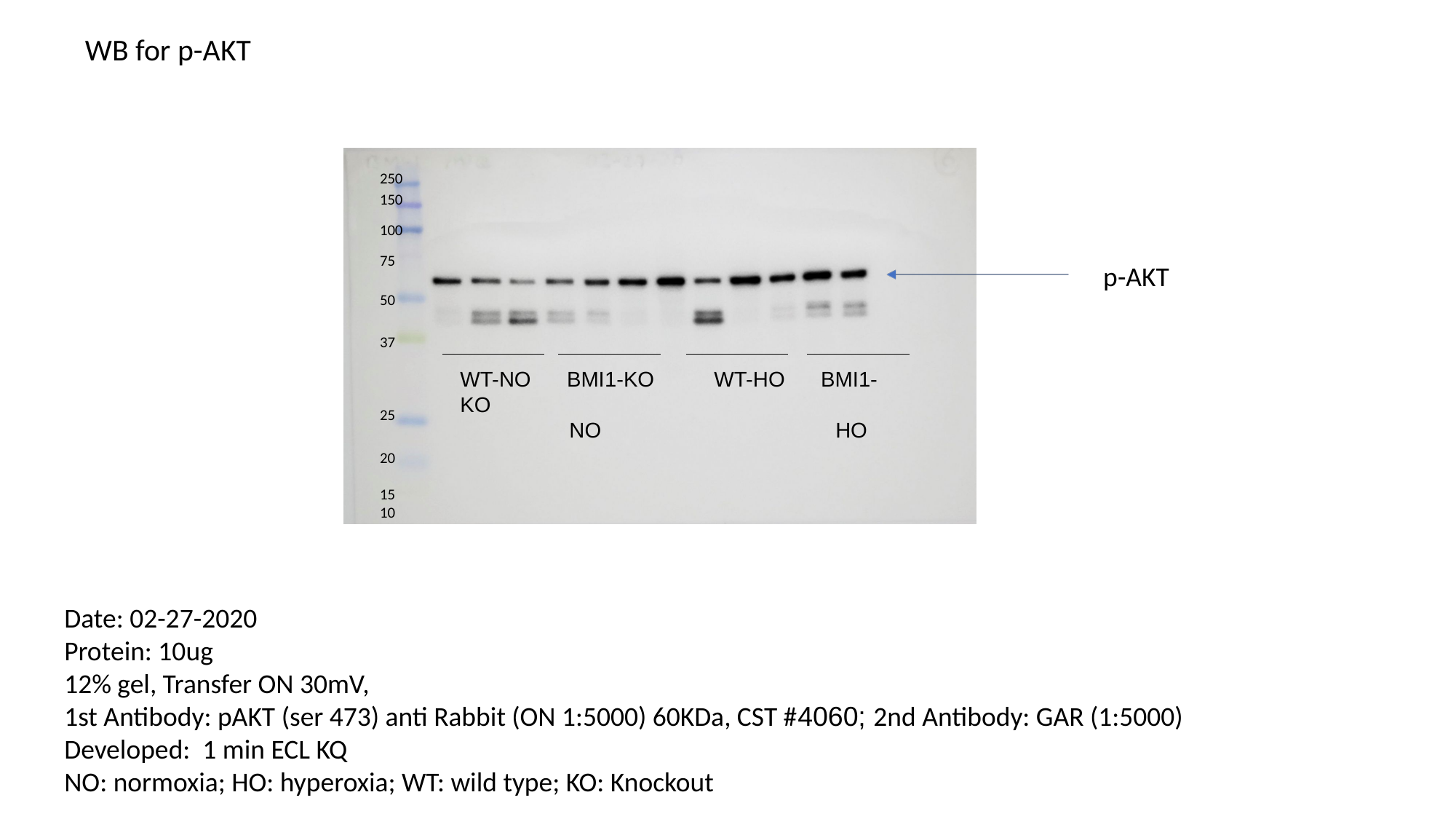

WB for p-AKT
250
150
100
75
50
37
25
20
15
10
p-AKT
WT-NO BMI1-KO WT-HO BMI1-KO
	NO 		 HO
Date: 02-27-2020
Protein: 10ug
12% gel, Transfer ON 30mV,
1st Antibody: pAKT (ser 473) anti Rabbit (ON 1:5000) 60KDa, CST #4060; 2nd Antibody: GAR (1:5000)
Developed: 1 min ECL KQ
NO: normoxia; HO: hyperoxia; WT: wild type; KO: Knockout

## Slide 8
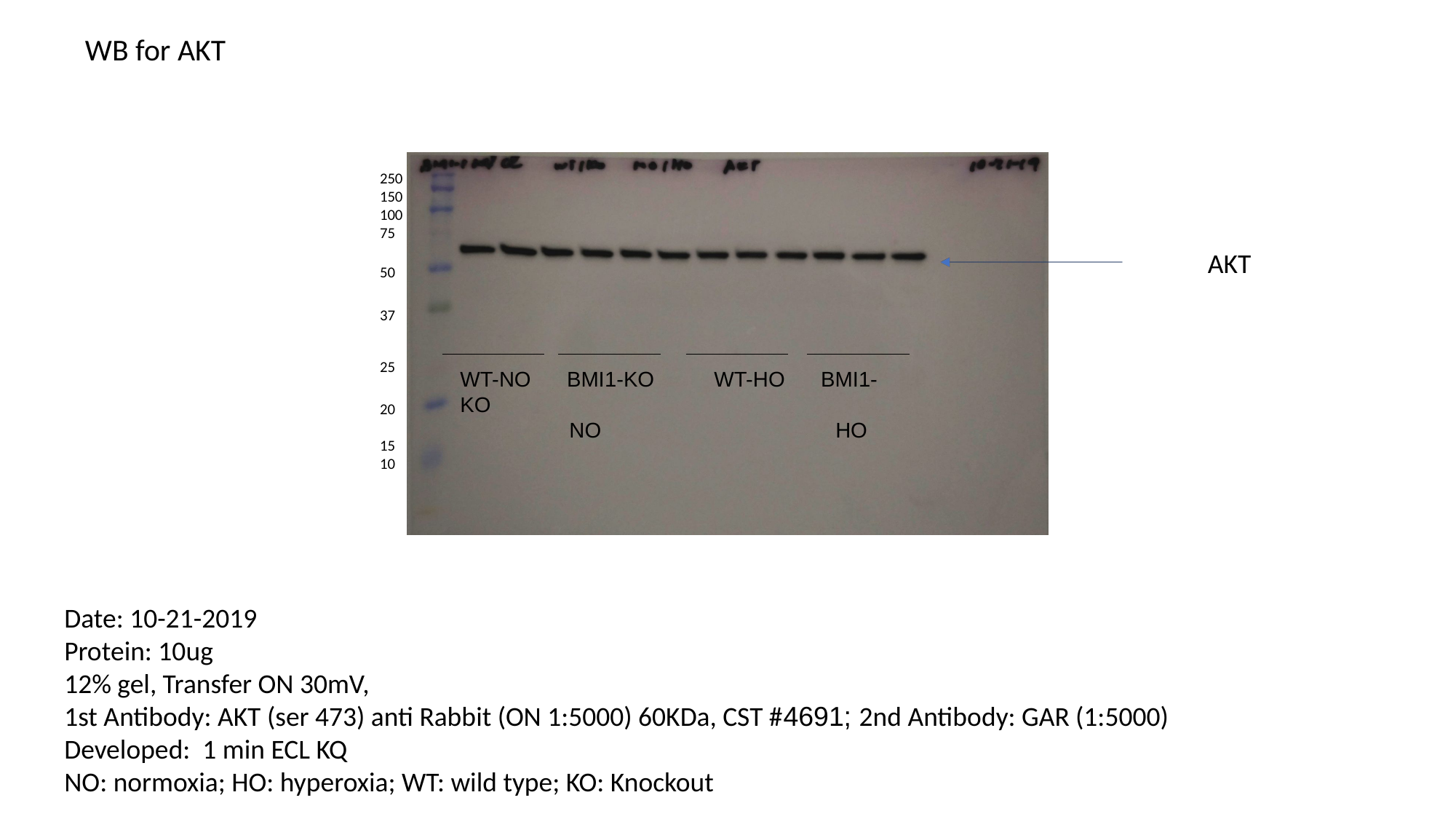

WB for AKT
250
150
100
75
50
37
25
20
15
10
AKT
WT-NO BMI1-KO WT-HO BMI1-KO
	NO 		 HO
Date: 10-21-2019
Protein: 10ug
12% gel, Transfer ON 30mV,
1st Antibody: AKT (ser 473) anti Rabbit (ON 1:5000) 60KDa, CST #4691; 2nd Antibody: GAR (1:5000)
Developed: 1 min ECL KQ
NO: normoxia; HO: hyperoxia; WT: wild type; KO: Knockout

## Slide 9
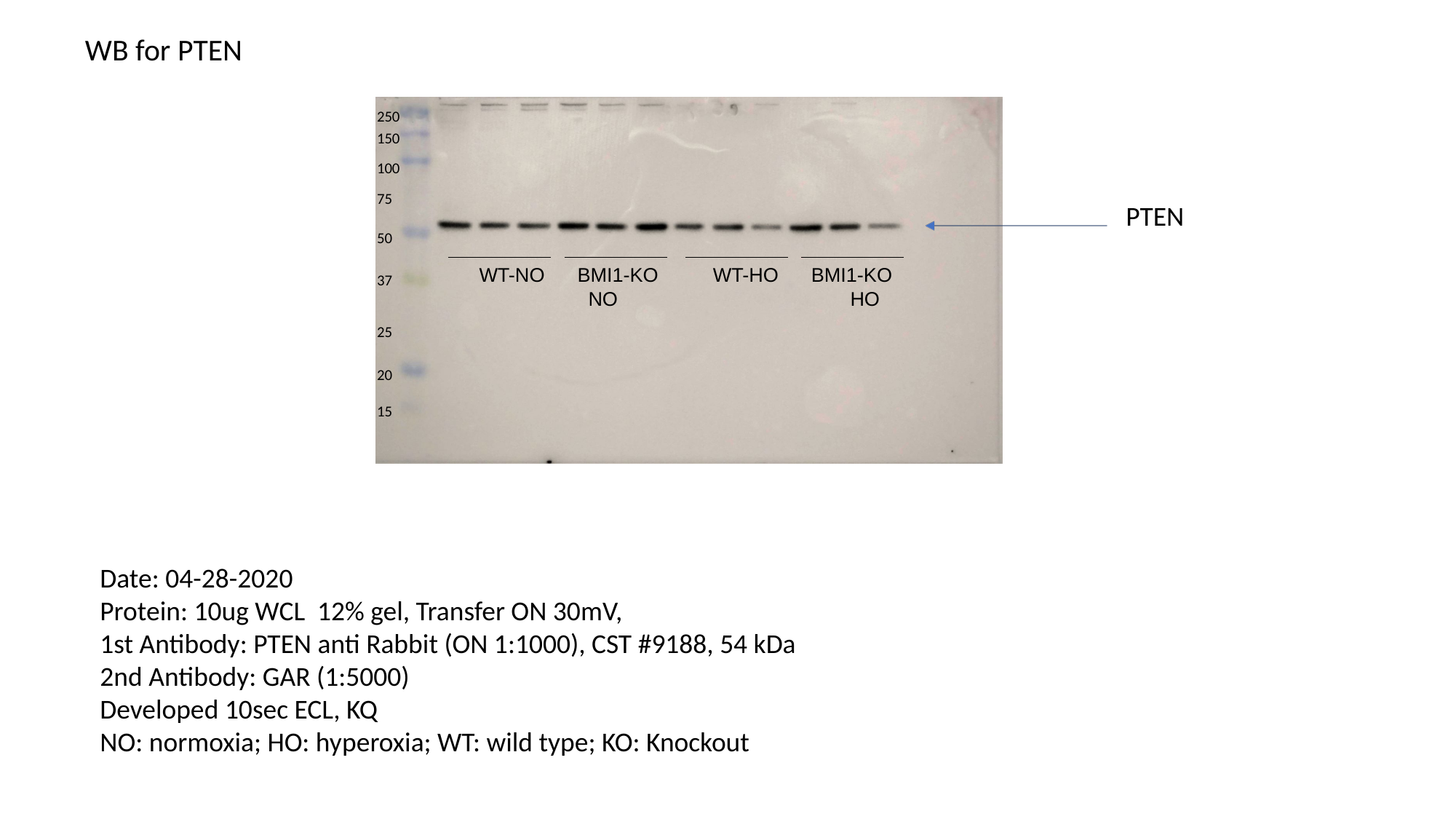

WB for PTEN
250
150
100
75
50
37
25
20
15
WT-NO BMI1-KO WT-HO BMI1-KO
	NO 		 HO
PTEN
Date: 04-28-2020
Protein: 10ug WCL 12% gel, Transfer ON 30mV,
1st Antibody: PTEN anti Rabbit (ON 1:1000), CST #9188, 54 kDa
2nd Antibody: GAR (1:5000)
Developed 10sec ECL, KQ
NO: normoxia; HO: hyperoxia; WT: wild type; KO: Knockout

## Slide 10
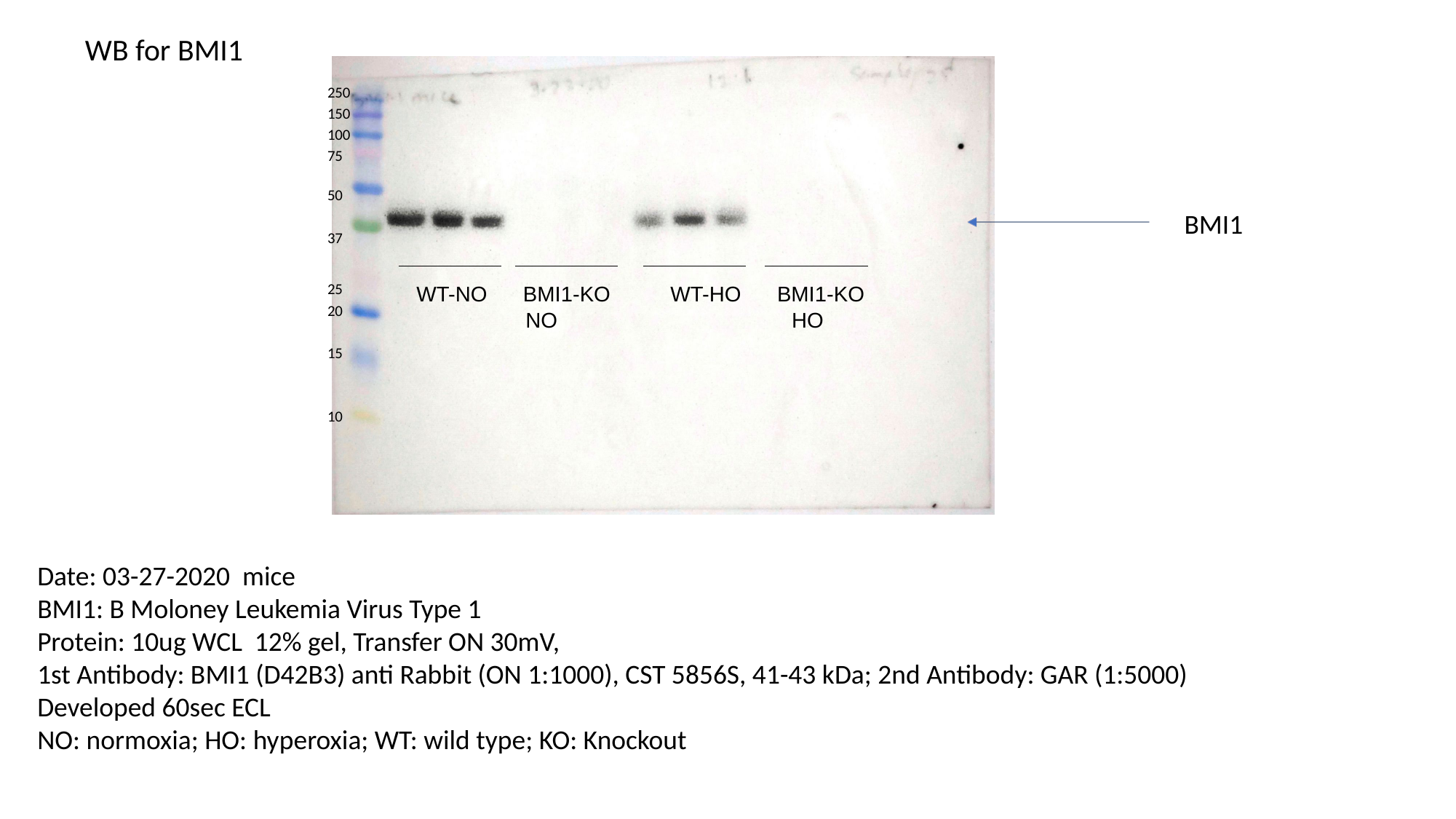

WB for BMI1
250
150
100
75
50
37
25
20
15
10
WT-NO BMI1-KO WT-HO BMI1-KO
	NO 		 HO
BMI1
Date: 03-27-2020 mice
BMI1: B Moloney Leukemia Virus Type 1
Protein: 10ug WCL 12% gel, Transfer ON 30mV,
1st Antibody: BMI1 (D42B3) anti Rabbit (ON 1:1000), CST 5856S, 41-43 kDa; 2nd Antibody: GAR (1:5000)
Developed 60sec ECL
NO: normoxia; HO: hyperoxia; WT: wild type; KO: Knockout

## Slide 11
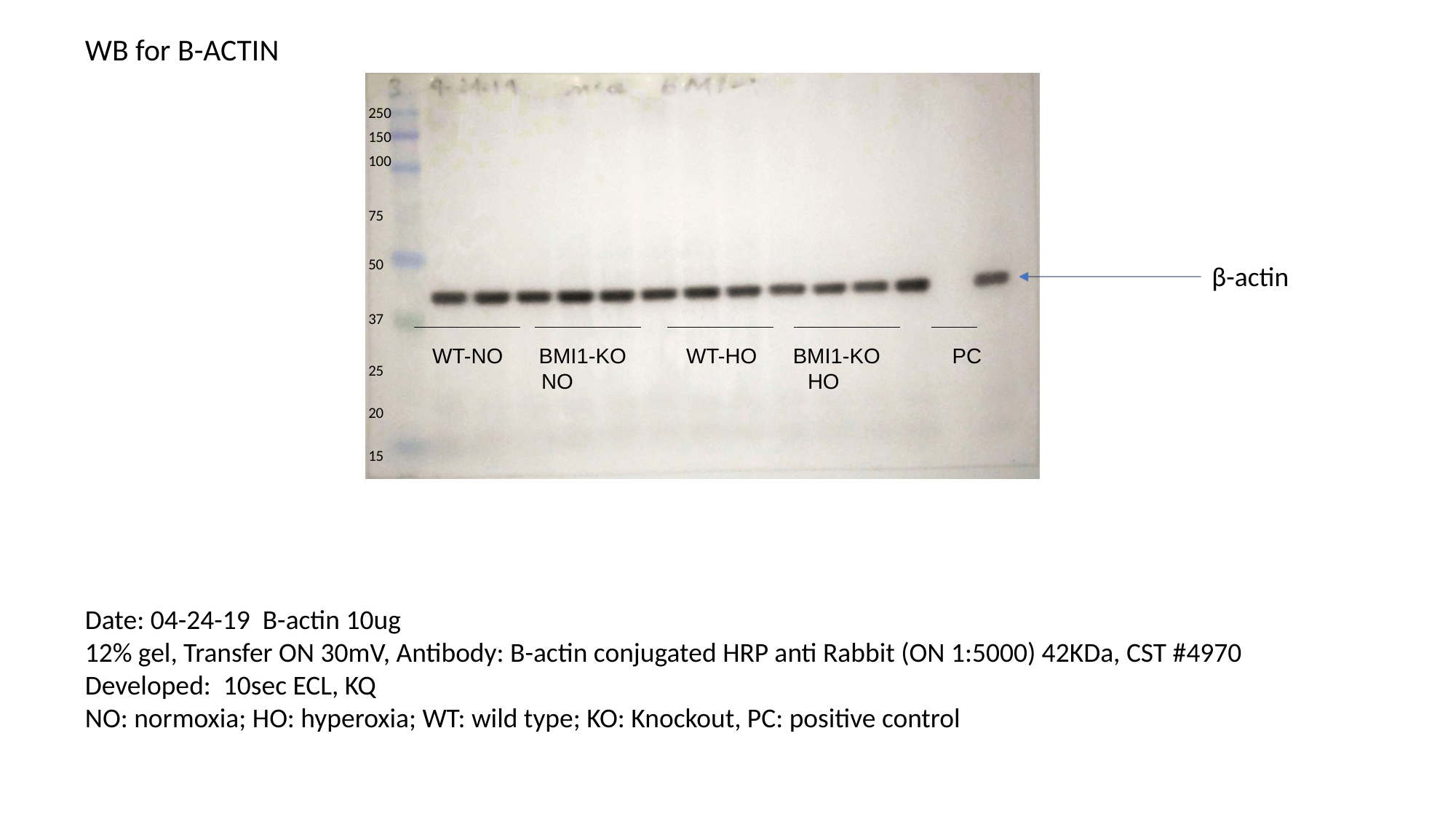

WB for B-ACTIN
250
150
100
75
50
37
25
20
15
β-actin
WT-NO BMI1-KO WT-HO BMI1-KO PC
	NO 		 HO
Date: 04-24-19 B-actin 10ug
12% gel, Transfer ON 30mV, Antibody: B-actin conjugated HRP anti Rabbit (ON 1:5000) 42KDa, CST #4970
Developed: 10sec ECL, KQ
NO: normoxia; HO: hyperoxia; WT: wild type; KO: Knockout, PC: positive control
