# Supplementary material for: Bmi1 deficiency exacerbates hyperoxia-induced acute lung injury in mice
Source: Front Physiol. 2025 Nov 13;16:1695456. doi: 10.3389/fphys.2025.1695456 (PMC12658778; doi:10.3389/fphys.2025.1695456)
Supplement: Supplementary file 12 [file Table2.docx]

### Supplemental Material

### Methods

### Reverse transcription-quantitative polymerase chain reaction (RT-qPCR)

Total RNA was isolated from lung tissue using RNeasy Mini Kit® from Qiagen (Germantown, MD), purified with RNase-free DNase set (Qiagen), dissolved in RNase-free water and stored at -80°C. A total of 1 μg of RNA was reverse transcribed using iScript cDNA synthesis Kit (BioRad, Hercules, CA) following the manufacturer’s instructions. To verify that knockout mice did not have the transcript for Bmi1, quantitative RT -qPCR was performed using cDNAs and TaqMan qRT-PCR probes: Bmi1(Mm00776122_gH), β-Actin (Mm02619580_g1). The qRT-PCR experiments were analyzed in a QuantStudio 3 PCR System (Life Technologies) according to the manufacturer’s instructions for TaqMan probes and TaqMan Fast Advanced Master Mix. Data were analyzed by ΔΔ*C*_t_ method using β-actin as an internal calibrator.
